# Nitrogen metabolism of two contrasting poplar species during acclimation to limiting nitrogen availability

**DOI:** 10.1093/jxb/ert234

**Published:** 2013-08-20

**Authors:** Jie Luo, Hong Li, Tongxian Liu, Andrea Polle, Changhui Peng, Zhi-Bin Luo

**Affiliations:** ^1^College of Life Sciences and State Key Laboratory of Crop Stress Biology in Arid Areas, Northwest A&F University, Yangling, Shaanxi 712100, PR China; ^2^Key Laboratory of Applied Entomology, College of Plant Protection, Northwest A&F University, Yangling, Shaanxi 712100, PR China; ^3^Büsgen-Institute, Department of Forest Botany and Tree Physiology, Georg-August University, Büsgenweg 2, 37077 Göttingen, Germany; ^4^Key Laboratory of Environment and Ecology in Western China of Ministry of Education, College of Forestry, Northwest A&F University, Yangling, Shaanxi 712100, PR China

**Keywords:** Gene expression, glutamate synthase, glutamine synthetase, net flux, nitrate reductase, nitrite reductase, plasma membrane H#x002B;-ATPase, poplar, stable carbon isotope.

## Abstract

To investigate N metabolism of two contrasting *Populus* species in acclimation to low N availability, saplings of slow-growing species (*Populus popularis*, Pp) and a fast-growing species (*Populus alba* × *Populus glandulosa*, Pg) were exposed to 10, 100, or 1000 μM NH_4_NO_3_. Despite greater root biomass and fine root surface area in Pp, lower net influxes of NH_4_^+^ and NO_3_^–^ at the root surface were detected in Pp compared to those in Pg, corresponding well to lower NH_4_^+^ and NO_3_^–^ content and total N concentration in Pp roots. Meanwhile, higher stable N isotope composition (δ^15^N) in roots and stronger responsiveness of transcriptional regulation of 18 genes involved in N metabolism were found in roots and leaves of Pp compared to those of Pg. These results indicate that the N metabolism of Pp is more sensitive to decreasing N availability than that of Pg. In both species, low N treatments decreased net influxes of NH_4_^+^ and NO_3_^–^, root NH_4_^+^ and foliar NO_3_^–^ content, root NR activities, total N concentration in roots and leaves, and transcript levels of most ammonium (*AMTs*) and nitrate (*NRTs*) transporter genes in leaves and genes involved in N assimilation in roots and leaves. Low N availability increased fine root surface area, foliar starch concentration, δ^15^N in roots and leaves, and transcript abundance of several *AMTs* (e.g. *AMT1;2*) and *NRTs* (e.g. *NRT1;2* and *NRT2;4B*) in roots of both species. These data indicate that poplar species slow down processes of N acquisition and assimilation in acclimation to limiting N supply.

## Introduction

As woody crops, forest plantations hold a great potential for the pulp and paper industry, carbon mitigation, and biomass production for biofuels (Luo *et al.*, 2006; Luo and Polle, 2009; Novaes *et al.*, 2009; Studer *et al.*, 2011). Plantations of some fast-growing tree species such as *Populus* spp. have been widely established in recent years (Weih, 2004; Polle and Douglas, 2010; Rennenberg *et al.*, 2010). As a riparian species, *Populus* in its natural habitat is supplied with sufficient nitrogen (N) derived from intensive N-fertilization application in agriculture (Rennenberg *et al.*, 2010; Koyama and Kielland, 2011). Due to the high demand of fertile soil for agriculture, however, poplar plantations have often been established on marginal lands where soil N is limiting (Rennenberg *et al.*, 2010; Bilodeau-Gauthier *et al.*, 2011). In this context, it is of particular importance to select poplar species with tolerance to low N availability.

The genus *Populus* contains about 30–40 species which may differ in N metabolism (Calfapietra *et al.*, 2007; Finzi *et al.*, 2007; Euring *et al.*, 2012; Li *et al.*, 2012). For instance, *Populus tremula* × *Populus tremuloides* is a species that often occurs on nutrient-poor soil. The growth and wood properties of this species are more responsive to different N levels than those of *Populus trichocarpa* which is adapted to fluctuating N supply (Euring *et al.*, 2012). These results highlight that it is essential to better understand the distinctness of N metabolism in different poplar species in order to select poplars with tolerance to low N availability.

Although little information is available on responses of N metabolism in different woody plants to low N availability, distinct N metabolism has been reported in different herbaceous species or genotypes in response to N deficiency (Lawlor *et al.*, 1987*a*, 1988, 1989; Lawlor, 2002; Hirel *et al.*, 2007; Shen *et al.*, 2013). For instance, some maize varieties displayed a higher capacity to absorb and utilize N than the others (Banziger *et al.*, 1997; Toledo Marchado and Silvestre Fernandes, 2001). In cereal crops, earlier studies demonstrated that N deficiency in soil often led to altered root length and branching and decreased soluble protein concentration and photosynthetic activity (Maizlich *et al.*, 1980; Lawlor *et al.*, 1987*b*, 1989; Lawlor, 2002; Hirel *et al.*, 2007). Plants with tolerance to low N are often associated with higher photosynthetic N use efficiency (PNUE), greater root length, and surface area per volume of soil (Lawlor, 2002; Hirel *et al.*, 2007; Shen *et al.*, 2013). However, the physiological and molecular mechanisms remain to be elucidated for plant species differing in tolerance to low N supply.

**Fig. 1.**
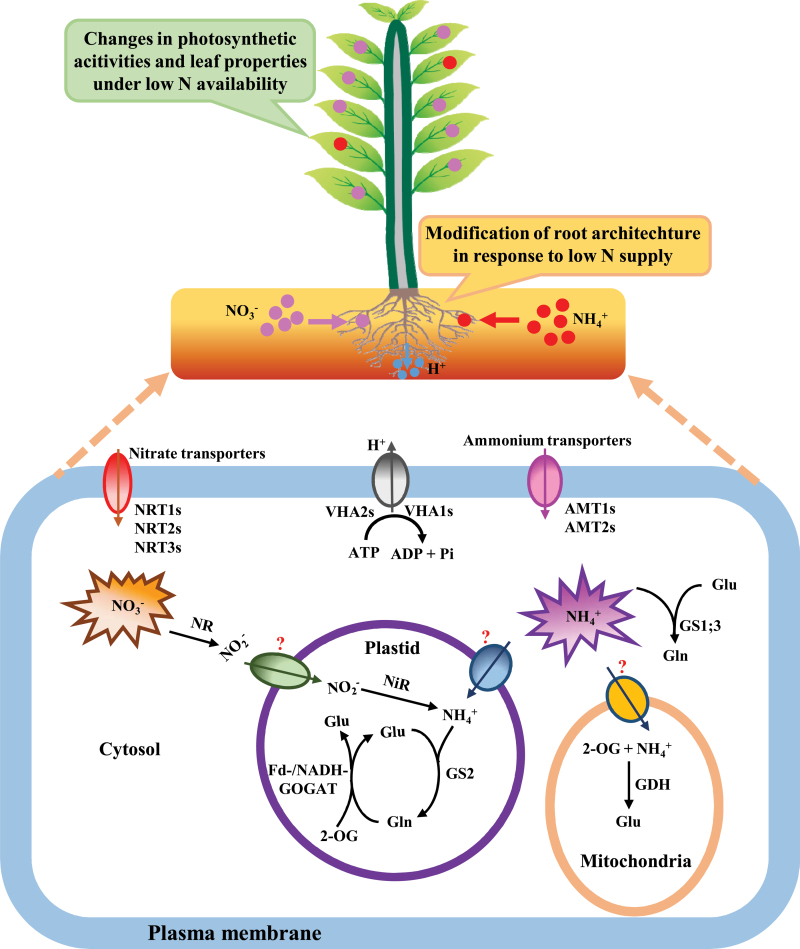
A conceptual model of N metabolism in plants. In the uptake process, NH_4_^+^ and NO_3_^–^ enter the cytosol via ammonium (AMTs) and nitrate (NRTs) transporters, respectively, coupled with plasma membrane H^+^-ATPases (VHAs). After uptake in roots, NH_4_^+^ and NO_3_^–^ can be translocated to leaves or other parts of the plant. In the assimilation process, NO_3_^–^ is converted to NH_4_^+^ by the cytosolic nitrate reductase (NR) and the plastidic/chloroplastic nitrite reductase (NiR). Subsequently, NH_4_^+^ can be assimilated to glutamine (Gln) catalysed by glutamine synthetase (GS) isoenzymes either in the plastid or the cytosol. The Gln in the plastid with 2-oxoglutarate (2-OG) can be further converted to glutamate (Glu) by Fd- or NADH-dependent glutamate synthase (Fd/NADH-GOGAT). Additionally, in the mitochondrion, NH_4_^+^ can be assimilated to Glu with glutamate dehydrogenase (GDH). The synthesized N compounds provide precursors for amino acids, proteins, and other N-containing metabolites which can be utilized by plant growth. At the cellular level, N metabolism in plants can be affected by external low N availability. At the plant level, root characteristics, photosynthetic activity, and leaf properties can be altered in response to low N supply (this figure is available in colour at *JXB* online).

In plants, the N metabolism process involves uptake, transport, assimilation, and utilization for amino acid biosynthesis and ultimately for growth (Nunes-Nesi *et al.*, 2010). Each of these steps may be regulated slightly different, leading to differences in N metabolism and performance of plants with distinct ecological requirements. In herbaceous plants, the N metabolism processes are well documented (Fig. 1), which may serve as a conceptual model to address N metabolism of different poplar species in response to low N availability.

In the N uptake process, NH_4_^+^ and nitrate NO_3_^–^ in soil solution are the two major inorganic N forms for plant absorption. Although both ions can be used by plants, the energetic, biochemical, and molecular features of NH_4_^+^ and NO_3_^–^ are different for metabolism, leading to distinct net fluxes of both ions at the root surfaces and NH_4_^+^ or NO_3_^–^ preference of plants (Jackson *et al.*, 2008; Patterson *et al.*, 2010). Using ^15^N labelling, it was demonstrated that some woody plants prefer NH_4_^+^ (Rennenberg *et al.*, 2009, 2010). Additionally, net fluxes of NH_4_^+^ and/or NO_3_^–^ at the root surfaces have been investigated using ion-selective microelectrodes in herbaceous and woody plants (Plassard *et al.*, 2002; Gobert and Plassard, 2007; Hawkins *et al.*, 2008; Hawkins and Robbins, 2010; Alber *et al.*, 2012; Luo *et al.*, 2013), providing a better understanding of electrophysiological processes of NH_4_^+^ and NO_3_^–^ acquisition. The net fluxes of both NH_4_^+^ and NO_3_^–^ are coupled with the activities of plasma membrane (PM) H^+^-ATPases in fine roots of *Populus popularis* (Luo *et al.*, 2013). However, comparisons of fluxes of NH_4_^+^ and NO_3_^–^ at the root surface of poplar species with large differences in growth are missing.

The fluxes of NH_4_^+^ and NO_3_^–^ are mediated by various transporters for ammonium (AMTs) and nitrate (NRTs) (Rennenberg *et al.*, 2010; Xu *et al.*, 2012). Some AMTs and NRTs have been functionally elucidated in *Arabidopsis thaliana* (Wang *et al.*, 2012; Xu *et al.*, 2012). For instance, transcript abundance of *AtAMT1;1* is strongly increased by N starvation and reduced upon NH_4_^+^ supply in *Arabidopsis* roots (Engelsberger and Schulze, 2012). Some NRT members such as AtNRT1;1, AtNRT1;2, AtNRT2;1, and AtNRT3;1 play pivotal roles in NO_3_^–^ uptake and signalling (Yong *et al.*, 2010; Kotur *et al.*, 2012; Wang *et al.*, 2012). In the genome of *P. trichocarpa*, 14 putative AMTs have been documented (Tuskan *et al.*, 2006; Couturier *et al.*, 2007), whereas less is known about the NRT members (Plett *et al.*, 2010; Rennenberg *et al.*, 2010; Li *et al.*, 2012). Several studies showed that transcripts of putative poplar transporters (e.g. AMT1;2, AMT1;6, AMT2;1, NRT1;1, NRT1;2, NRT2;4B, NRT2;4C, NRT3.1B, and NRT3.1C) were responsive to environmental fluctuations under non-limiting N conditions (Selle *et al.*, 2005; Couturier *et al.*, 2007; Dluzniewska *et al.*, 2007; Ehlting *et al.*, 2007; Plett *et al.*, 2010; Li *et al.*, 2012). However, it remains unknown how transcriptional regulation of these transporters responds to limiting N supply in different poplar species.

After uptake into the roots, a large amount of NH_4_^+^ can be assimilated locally and the remainder is translocated to leaves or other parts of the plant, whereas only a limited amount of NO_3_^–^ is assimilated in roots and most NO_3_^–^ is transported to leaves (Black *et al.*, 2002; Xu *et al.*, 2012). In the assimilation process, NO_3_^–^ is converted to NH_4_^+^ by nitrate reductase (NR) and nitrite reductase (NiR) (Xu *et al.*, 2012). Subsequently, NH_4_^+^ can be assimilated to glutamine catalysed by glutamine synthetase (GS) (Castro-Rodriguez *et al.*, 2011; Coleman *et al.*, 2012). The formation of glutamate requires glutamine and 2-oxoglutarate in a reaction catalysed by glutamate synthase (GOGAT) (McAllister *et al.*, 2012). Additionally, glutamate can be synthesized by glutamate dehydrogenase (GDH) under consumption of NH_4_^+^ and 2-oxoglutarate (McAllister *et al.*, 2012). Very little is known about the response of these enzymes to low N supply in different poplar species.

Although forest plantations often grow on nutrient-poor soils (Johnson, 2006; Rennenberg *et al.*, 2009), N-related studies in trees mainly addressed the effects of fertilization but less to uncover the responses to N-limitation (Cooke and Weih, 2005; Rennenberg *et al.*, 2009, 2010; Lukac *et al.*, 2010; Millard and Grelet, 2010). Recently, we found that growth, carbon, and N physiology, and wood properties of the fast-growing *Populus alba* × *Populus glandulosa* (Pg), which generally grows on relatively fertile soils, displays a stronger responsiveness to N-fertilization than the slow-growing *P. popularis* (Pp) which is often found on nutrient-deficient soils (Li *et al.*, 2012). The differential responses are ascribed to prioritized resource allocation to the leaves and accelerated N physiological processes in the fast-growing Pg under higher N supply levels. However, it remains unknown how N metabolism processes and key components involved in these processes of Pp and Pg respond to external low N availability.

This study exposed Pp and Pg to low N levels. Measure ments of morphological (root characteristics), physiological (e.g. photosynthesis, net fluxes of NH_4_^+^, NO_3_^–^, and H^+^, accumulation of NH_4_^+^, NO_3_^–^, and NO_2_^–^, total N concentration, and δ^15^N), and molecular (transcript levels of representative genes involved in N metabolism) parameters known to be important for acclimation to low N availability were conducted. Furthermore, multivariate analysis was applied to dissect the importance of parameters as contributors to the acclimation of N metabolism in both poplar species to low N supply. The following questions were specifically addressed: (i) do root morphology, photosynthesis, and N metabolism of Pp and Pg display different response patterns to limiting N supply? and (ii) what are the physiological and transcriptional regulation mechanisms of Pp and Pg in acclimation to low N availability?

## Materials and methods

### Plant cultivation and N treatment

Cuttings of the slow-growing *P. popularis* (Pp) and the fast-growing *P. alba × P. glandulosa* (Pg) were rooted as described previously (Li *et al.*, 2012), and planted in pots (10 l) filled with fine sand. Plants were cultivated in a greenhouse (natural light, day/night 25/20 °C, 75% relative humidity) and provided with 50ml Long Ashton (LA) nutrient solution, which contains 1000 μM NH_4_NO_3_ (Dluzniewska *et al.*, 2007) every other day. After 6 weeks, plants with similar height (*c.*60cm) were selected for further study. The root systems of selected plants were carefully washed with tap water. Plants of each species were divided into three groups with 18 plants for each group. Subsequently, plants of three groups from each species were cultivated in hydroponics with modified LA solution (0.5mM KCl, 0.9mM CaCl_2_, 0.3mM MgSO_4_, 0.6mM KH_2_PO_4_, 42 μM K_2_HPO_4_, 10 μM Fe-EDTA, 2 μM MnSO_4_, 10 μM H_3_BO_3_, 7 μM Na_2_MoO_4_, 0.05 μM CoSO_4_, 0.2 μM ZnSO_4_, and 0.2 μM CuSO_4_) containing 10, 100, or 1000 μM NH_4_NO_3_, respectively, and the nutrient solution was adjusted to pH 5.5. The LA solution was refreshed every 2 days. In the greenhouse, the position of each plant was randomly assigned and altered once a week. At the beginning of the N treatment, the apex of each plant was marked by using a laboratory marker to distinguish the shoots formed during the N treatment. After hydroponic cultivation with N treatments for 3 weeks from 20 June to 10 July, 12 plants of each group were used for gas exchange determination prior to harvest and the remaining six plants of each group were used for measurements of net fluxes of NH_4_^+^ and NO_3_^–^.

### Gas exchange and harvesting

The gas exchange of three mature leaves (leaf plastochron index = 8–10) formed during N treatment was determined for each plant. Net photosynthetic rates (*A*), stomatal conductance (*g*_s_), and transpiration rates were determined with a portable photosynthesis system (Li-Cor-6400, Li-Cor, Lincoln, NE, USA) and an attached LED light source (6400–02) as described by He *et al.* (2011). The instantaneous photosynthetic N use efficiency (PNUE_i_) was calculated based on *A*, foliar N concentration, and specific leaf area, as suggested by Li *et al.* (2012). As a stored photosynthate, starch concentration in harvested root and leaf tissues (see below) was analysed as suggested by He *et al.* (2013).

Since the daily rhythm of plants can affect physiological and molecular processes (Wilkins *et al.*, 2009), the harvest was performed between 9:00 and 12:00. For harvest, the root system of each plant was well washed with corresponding LA solution containing 10, 100, or 1000 μM NH_4_NO_3_. Subsequently, the root system was wrapped in lab tissue paper to remove water on the root surface. Length of main root and fresh weight of roots were recorded. A part of roots (*c.*2g) of each plant was excised from the root system, scanned, and analysed by a WinRHIZO root analyser system (WinRHIZO version 2012b, Regent Instruments Canada, Montreal, Canada) as described by Luo *et al.* (2013). Leaves formed during the N treatment (above the mark on the stem) were harvested. Leaf discs for determination of specific leaf area were also collected and specific leaf area was calculated according to the method of Cao *et al.* (2012). Harvested roots or leaves were wrapped with tinfoil and immediately frozen in liquid N. Root or leaf samples were ground into fine powder in liquid N with a mortar and pestle and stored at –80 °C. Frozen powder (*c.*100mg) from roots or leaves of each plant was dried at 60 °C for 72h to determine the fresh-to-dry-mass ratio. For further biochemical analysis, equal weight of fine powder from roots or leaves of two plants within each group was combined to form a well-mixed sample.

### Analysis of net fluxes of NH_4_^+^, NO_3_^–^, and H^+^

To analyse net fluxes of NH_4_^+^, NO_3_^–^, and H^+^ at the root surface, three white fine roots (*c.*1.5mm in diameter) were randomly selected from the root system of each plant. Net fluxes of these ions were measured non-invasively using scanning ion-selective electrode technique (SIET, SIET system BIO-003A, Younger USA Science and Technology, Falmouth, MA, USA) by Xuyue Science and Technology (Beijing, China). The SIET system and its application in net ion flux detection were described in detail (Li *et al.*, 2010; He *et al.*, 2011; Luo *et al.*, 2013). The ion-selective microelectrode with 2–4 μm aperture was manufactured and silanized with a backfilling solution and an ion-selective liquid cocktail.

To find out the positions along the root where the maximal net fluxes of NH_4_^+^ and NO_3_^–^ take place, a preliminary experiment was performed using plants treated with 100 μM NH_4_NO_3_ by taking an initial measurement at the root apex, followed by measurements at 300 μm intervals (in the region of 0–2100 μm) or 5mm intervals (in the region of 5–30mm) along the root tip (Supplementary Fig. S1A, available at *JXB* online). Ion gradients near the root surface were measured by moving the ion-selective microelectrode between two positions (*c.*30 μm in distance) in a perpendicular direction to the root axis. The recording rate for these ion fluxes was one reading per 6 s and ion flux was recorded at each measurement point for 10min.

For the positions where the maximal net fluxes of NH_4_^+^ and NO_3_^–^ occur in roots, net fluxes of NH_4_^+^, NO_3_^–^, and H^+^ were further investigated in detail. A fine root was transferred to a Petri dish containing 10ml measuring solution (0.1mM KCl, 0.1mM CaCl_2_, pH 5.5) with 10, 100, or 1000 μM NH_4_NO_3_ according to the N treatment of the selected root, and equilibrated for 20min. Prior to the measurement, the root was transferred to a new Petri dish containing fresh measuring solution and net NH_4_^+^ fluxes were monitored for 10min. Afterwards, net NO_3_^–^ fluxes were measured for 10min in the same root and finally, net H^+^ fluxes were recorded for 10min in the root.

### Isolation of the PM and measurement of PM H^+^-ATPase activity

PM vesicles of root cells were isolated according to the method of Sorgona *et al.* (2011) with minor modification. Briefly, fine powder of roots (*c.*2g) was homogenized with 3ml extraction solution containing 250mM sucrose, 10% (v/v) glycerol, 10mM glycerol-1-phosphate, 2mM MgSO_4_, 2mM EDTA, 2mM EGTA, 2mM ATP, 2mM DTT, 5.7% (w/v) choline chloride, 25mM 1,3-bis(tris (hydroxymethyl)-methyl-aminoethylether)propane (BTP, pH 7.6 with 2(N-morpholino)ethanesulphonic acid, MES), 1mM PMSF, and 20 μg ml^–1^ chimostatin at 4 °C. After centrifugation (12,700 *g*, 4 °C, 30min), the pellets were suspended over a 25/38% discontinuous sucrose gradient (5mM MES containing all protectants present in the extraction solution, pH 7.4). Afterwards, the gradient was centrifuged again (12,700 *g*, 4 °C, 60min). The pellets were resuspended in a medium containing 20% glycerol (v/v), 2mM EGTA, 2mM EDTA, 0.5mM ATP, 1mM PMSF, 2mM DTT, 20 μg ml^–1^ chimostatin, 5.7% choline chloride, and 5mM BTP buffered at pH 7.0 with MES and immediately frozen in liquid N and stored at –80 °C.

PM H^+^-ATPase activity was determined spectrophotometrically at 700nm as described by Sorgona *et al.* (2011) with minor modifications. In brief, assays were carried out at 30 °C in 0.5ml medium containing 30mM BTP/MES (pH 6.5), 5mM MgSO_4_, 50mM KCl, 4mM ATP, 0.6mM Na_2_MoO_4_, 100mM KNO_3_, 1.5mM NaN_3_, and 0.02% (w/v) polyxyethylene 20 cetyl ether, with or without 100 μM vanadate (an inhibitor of P-type H^+^-ATPase). The difference between these two activities was attributed to the PM H^+^-ATPase. Sodium azide and KNO_3_ were used as selective inhibitors of mitochondrial and tonoplast H^+^-ATPase, respectively (Zhu *et al.*, 2009). The reaction was initiated by adding membrane vesicles (5–10 μg membrane protein) and stopped after 30min with a solution containing 2% (v/v) concentrated H_2_SO_4_, 5% (w/v) SDS, 0.7% Na_2_MoO_4_, and 10% ascorbic acid. After solubilizing the membrane vesicles with 0.5M NaOH (Gogstad and Krutnes, 1982), the total soluble protein was estimated according to Bradford (1976). PM H^+^-ATPase activity was expressed as that inhibited by 100 μM vanadate.

### Determination of NH_4_^+^, NO_3_^–^, and NO_2_^–^ concentration

NH_4_^+^ concentration in roots and leaves was determined based on the Berthelot reaction (Brautigam *et al.*, 2007; Luo *et al.*, 2013). In brief, fine power (*c.*100mg) was homogenized in an extraction solution (1ml 100mM HCl and 500 μl chloroform). The extraction solution was centrifuged (10,000 *g*, 4 °C, 10min) after shaking for 15min at 4 °C. The aqueous phase was transferred to a 2ml tube with 50mg activated charcoal, mixed well, and centrifuged (12,000 *g*, 4 °C, 5min) again. NH_4_^+^ concentration in the supernatant was determined spectrophotometrically at 620nm.

NO_3_^–^ concentration in samples was analysed as suggested by Patterson *et al.* (2010). Fine powder (*c.*100mg) was extracted in 1ml deionized water at 45 °C for 1h. After centrifugation (5000 *g*, 20 °C, 15min), the supernatant was used for nitrate quantification. The supernatant (0.2ml) was mixed thoroughly with 0.8ml of 5% (w/v) salicylic acid in concentrated H_2_SO_4_. After incubation at room temperature for 20min, 19ml of 2M NaOH was added to raise the pH to above 12. The solution was cooled to room temperature before NO_3_^–^ concentration was determined spectrophotometrically at 410nm.

NO_2_^–^ concentration in samples was quantified as described by Ogawa *et al.* (1999). Frozen fine powder (*c.*100mg) was extracted by an extraction buffer containing 50mM TRIS-HCl (pH 7.9), 5mM cysteine, and 2mM EDTA. After centrifugation (10,000 *g*, 20 °C, 20min), 500 μl supernatant was mixed with 250 μl 1% sulphanilamide and 250 μl 0.02% N-(1-naphtyl)-ethylene-diamine dihydrochloride in 3.0M HCl. NO_2_^–^ concentration was quantified spectrophotometrically at 540nm.

### Determination of enzyme activities

Activities of NR (EC 1.7.99.4) and NiR (EC 1.7.2.1) were determined in roots and leaves according to the methods of Ehlting *et al.* (2007) and Ogawa *et al.* (1999), respectively. Briefly, frozen powder (*c.*200mg) was extracted in an extraction buffer (100mM HEPES-KOH (pH 7.5), 5mM Mg-acetate, 5mM DTT, 1mM EDTA, 0.5mM PMSF, 20mM FAD, 5mM Na_2_MoO_4_, 10% (v/v) glycerin, 1% (w/v) polyvinyl polypyrrolidone, 0.5% BSA, 0.1% (v/v) TritonX-100, and either 25mM leupeptine for leaves or 25mM chymostatine for roots). The crude extract was used for NR and NiR assays.

For NR, the extract was added to the reaction mixture (100mM HEPES-KOH (pH 7.5), 6.0mM KNO_3_, 6.0mM EDTA, 0.6mM NADH, 12mM FAD, 6mM Na_2_MoO_4_, 3mM DTT, and either 25mM leupeptine for leaves or 25mM chymostatine for roots) at 25 °C. The reaction was terminated after 20min by adding 0.6M Zn-acetate and 0.25mM phenazinemethosulphate. NO_2_^–^ formation in the solution was determined as in the assay of NO_2_^–^ concentration.

For NiR, 500 μl supernatant from NO_2_^–^ concentration assay was concentrated with a Amicon Ultra 10K filter (Millipore, Billerica, USA) to reduce nitrate ions. The concentrated supernatant was mixed with 500 μl solution containing 50mM TRIS-HCl (pH 7.5), 1mM cysteine, and 2mM EDTA. The NiR activity was determined by following the reduction of NO_2_^–^ in the assay. The assay solution contained 0.5mM NaNO_2_, 1mM methyl viologen, and the extract. The reaction was started by adding the reagent (0.12M Na_2_S_2_O_4_, 0.2M NaHCO_3_), incubated at 30 °C for 60min, and terminated by vigorous vortex until the colour of the methyl viologen disappeared completely. After adding 1M Zn-acetate, the mixture was centrifuged (10,000 *g*, 25 °C, 10min). The residual NO_2_^–^ in the reaction solution was determined as in the assay of NO_2_^–^ concentration.

GS (EC 6.3.1.2) activity was analysed spectrophotometrically as proposed by Wang *et al.* (2008). Frozen fine powder was homogenized at 4 °C in 50mM TRIS-HCl extraction solution (pH 8.0) containing 2mM MgCl_2_, 2mM DTT, and 0.4M sucrose. After centrifugation (15,000 *g*, 4 °C, 20min), the supernatant was used for GS activity assay. The assay solution contained 0.35ml of 40mM ATP and 0.8ml of 0.1M TRIS-HCl buffer (pH 7.4) with 20mM Na-glutamate, 80mM MgSO_4_, 20mM cysteine, 2mM EGTA, and 80mM NH_2_OH. After adding the enzyme extract to the assay solution, the mixture was incubated (37 °C, 30min) and the incubation was stopped by addition of the reagent (0.37M FeCl_3_, 0.2M trichloroacetic acid, 0.6M HCl). After centrifugation (5000 *g*, 4 °C, 15min), the absorbance of the supernatant was recorded at 540nm. GS activity was expressed as 1 μmol γ-glutamyl hydroxamate formed per min.

Activities of GOGAT (EC 1.4.7.1) and GDH (EC 1.4.1.2) were assayed in roots and leaves based on the method of Lin and Kao (1996). Fine powder (*c.*100mg) was extracted with 10mM TRIS-HCl buffer (pH 7.6), 1mM MgC1_2_, 1mM EDTA, and 1mM β-mercaptoethanol at 4 °C. After centrifugation (15,000 *g*, 4 °C, 30min), the supernatant was used for determination of enzyme activities. The GOGAT assay solution contained 0.2ml of 20mM l-glutamine, 25 μl of 0.1M 2-oxoglutarate, 50 μl of 10mM KCI, 0.1ml of 3mM NADH, and 0.25ml enzyme extract in a final volume of 1.5ml made up with 25mM TRIS-HCl buffer (pH 7.6). After addition of l-glutamine, the decrease in absorbance was recorded spectrophotometrically at 340nm. The GDH assay mixture contained 0.15ml of 0.1M 2-oxoglutarate, 0.15ml of 1M NH_4_Cl, 0.1ml of 3mM NADH, and 0.5ml of the enzyme extract in a final volume of 1.5ml made up with 0.2M TRIS-HCl buffer (pH 8.0). After addition of enzyme extract, the decrease in absorbance was monitored spectrophotometrically at 340nm.

### Determination of total carbon and nitrogen and stable isotopes

Root samples and mature leaves used for gas exchange measurements were harvested for total C and N and stable isotope (^13^C and ^15^N) analysis. Total C and N concentration was determined according to the method of Luo and Polle (2009). The stable C isotope (^13^C) was analysed based on the protocol of Cao *et al.* (2012). Fine powder (*c.*50mg) was dried in an oven at 80 °C. The dried powder (*c.*0.8mg) was sealed under vacuum in a quartz tube with copper oxide and silver foil and combusted for at least 4h at 800–850 °C. The CO_2_ from the combustion tube was extracted and purified cryogenically. The isotopic ratio of the extracted CO_2_ was determined by an elemental analyser (NA 1110, CE Instruments, Rodano, Italy) and a mass spectrometer (Delta Plus, Finnigan MAT, Bremen, Germany) with an interface (Conflo III, Finnigan MAT, Bremen, Germany) according to the method of Werner *et al.* (1999). The ^13^C/^12^C ratio is expressed as parts per thousand deviation from the Pee Dee Belemnite standard. Carbon isotope composition (%) was calculated as δ^13^C = (R_sa_ – R_sd_)/R_sd_ × 1000, where R_sa_ and R_sd_ are the ratios of ^13^C to ^12^C of the sample and the standard, respectively. The standard was referred to CO_2_ in air.

Stable N isotope composition (δ^15^N) was analysed similarly to ^13^C with minor modifications, according to the method of Yousfi *et al.* (2012). The standard was referred to N_2_ in air.

### Determination of mineral nutrients, soluble sugars, soluble protein, and phenolics

Mineral elements in roots and leaves were analysed by an inductively coupled plasma-atomic emission spectrometer (Spectroflame, Spectro Analytical Instruments) based on the protocol of Heinrichs *et al.* (1986).

Concentrations of total soluble sugar in root and leaf tissues were analysed by the anthrone method of Yemm and Willis (1954) with minor modifications (He *et al.*, 2013). The standard curve was established by using a serial of diluted solutions of glucose. The final absorbance of total soluble sugar and starch (expressed as glucose equivalent) in samples was determined at 620nm.

Soluble protein in plant materials was extracted and used for quantification (Bradford, 1976). Soluble phenolics were determined according to the method of Swain and Goldstein (1964) with themodification by Luo *et al.* (2008).

### Analysis of transcript levels of genes involved in N uptake and assimilation

The transcriptional changes of genes implicated in N uptake and assimilation were analysed by reverse-transcription quantitative PCR (qPCR) based on the method (Li *et al.*, 2012). Briefly, frozen fine powder of roots (*c.*100mg) and leaves (*c.*50mg) was used for total RNA isolation. Total RNA was isolated and purified with a plant RNA extraction kit (R6827, Omega Bio-Tek, GA, USA) and trace genomic DNA was digested by DNase I (E1091, Omega Bio-Tek) attached to the RNA extraction kit. The lack of trace genomic DNA in total RNA was confirmed by a control PCR using total RNA as templates. Aliquots of 1 μg total RNA were used for first-strand cDNA synthesis using a PrimeScript RT reagent kit (DRR037S, Takara, Dalian, China) in a 20 μl reaction. In this reaction system, random primers and oligo dTs were added according to the manufacturer’s instructions. Real-time PCR was performed in a 20 μl reaction using 10 μl 2× SYBR Green Premix Ex Taq II (DRR081A, Takara), 2.5 μl cDNA, and 0.2 μl of 20mM primers (Supplementary Table S1) in an IQ5 Real Time System (Bio-Rad, Hercules, CA, USA). To ensure the primer specificity, PCR products were sequenced and aligned with homologues from *P. trichocarpa* and other model plants (Supplementary Fig. S2). *Actin2/7* was used as a reference gene (Brunner *et al.*, 2004). PCR was performed in triplicate together with a dilution series of the reference gene. The efficiencies of all PCR reactions were between 95 and 105% (Supplementary Table S1).

### Statistical analysis

Net flux data were calculated by Mageflux version 1.0 attached to the SIET system (Xu *et al.*, 2006). Statistical tests were performed with Statgraphics (STN, St Louis, MO, USA). Data were tested for normality before further analysis. The effects of species and N treatment on variables were analysed by two-way ANOVAs. Differences between means were considered significant when the *P*-value of the ANOVA F-test was less than 0.05. The Cq values obtained from qPCR were normalized using the program proposed by Pfaffl *et al.* (2002) and the fold-changes of transcript levels were calculated in the program of REST (Pfaffl *et al.*, 2002) as described by Li *et al.* (2012). For principal component analysis (PCA), data were standardized and computed by the command prcomp() in R (http://www.r-project.org/). The cluster analysis of gene expression was computed by command heatmap.2() with the package ‘gplots’ in R.

## Results

### Root morphology and photosynthesis

Root morphology and photosynthesis are sensitive to changes in resource (e.g. N) availability. Thus, these characteristics were analysed in both poplar species (Table 1). Pp and Pg had distinct root morphological characteristics under either the control N (1000 μM NH_4_NO_3_) or low N (100 and 10 μM NH_4_NO_3_) supply levels. Generally, Pg exhibited lower root biomass, total fine root length, and total fine root surface area, but higher root volume than Pp. Photosynthesis showed no differences between Pp and Pg under the control N level. Intrinsic water use efficiency was lower in Pg than that in Pp under the given N levels. As a stored photosynthate, foliar starch concentration was higher in Pg than those in Pp under the three N supply levels. Root starch concentration was similar in Pp and Pg.

**Table 1.** *Root morphological, photosynthetic characteristics, and starch of* P. popularis *(Pp) and* P. alba × P. glandulosa *(Pg) exposed to 10, 100, or 1000 µM NH*_*4*_ *NO*_*3*_

Species	N treatment (μM)	Root biomass (g DW)	Total fine root length (m)	Total fine root surface area (cm^2^)	Total root volume (cm^3^)	*A* (mmol CO_2_ m^–2^ s^–1^)	WUE_i_ (mmol CO_2_ mol^–1^ H_2_O)	PNUE_i_ (mol CO_2_ (mg N)^–1^ s^–1^)	Foliar starch (mg (g DW)^–1^)	Root starch (mg (g DW)^–1^)
Pp	10	4.4±0.3^bc^	21.9±2.0^a^	123.3±14.4^cd^	4.9±0.0^a^	6.8±0.7^a^	134.1±26.8^d^	1.8±0.1^a^	11.8±0.1^b^	12.3±0.8^ab^
	100	4.5±0.3^c^	39.9±2.2^b^	158.2±18.5^d^	4.6±0.8^a^	9.8±0.3^b^	120.7±3.2^d^	2.4±0.2^b^	11.2±1.2^b^	13.6±0.2^b^
	1000	3.5±0.3^abc^	19.6±2.1^a^	87.8±1.8^bc^	6.2±0.5^a^	17.1±0.8^cd^	73.7±0.2^c^	4.0±0.4^c^	8.3±0.1^a^	10.8±1.1^a^
Pg	10	2.5±0.4^a^	22.0±8.5^a^	71.2±11.8^ab^	5.7±0.7^a^	7.7±0.4^a^	40.9±0.2^a^	2.5±0.6^b^	14.0±0.1^c^	13.3±0.2^b^
	100	3.4±0.6^a^	11.5±1.8^a^	46.3±8.4^a^	9.2±0.5^b^	10.2±0.2^b^	45.2±1.8^ab^	2.8±0.5^bc^	10.9±0.5^b^	12.4±0.4^ab^
	1000	2.5±0.3^ab^	12.8±2.2^a^	48.9±7.5^a^	9.1±0.4^b^	14.6±0.7^c^	52.3±4.0^b^	3.3±0.2^c^	11.4±0.1^b^	12.0±0.9^ab^
*P*-values	Species	***	**	****	****	ns	****	ns	**	ns
	N	Ns	ns	*	**	***	ns	**	***	ns
	Species × N	Ns	**	*	*	*	*	ns	*	ns

Data indicate mean ± SE (*n* = 6). Different letters in the same column indicate significant difference (*P* < 0.05). *P*-values of the ANOVAs of species, N treatment, and their interaction are indicated: *P < 0.05; **P < 0.01; ***P < 0.001; ****P < 0.0001; ns, not significant. Pp, *Populus popularis*; Pg, *Populus alba* × *Populus glandulosa*; *A*, leaf net photosynthetic rate; PNUE_i_, instantaneous photosynthetic N use efficiency; WUE_i_, intrinsic water use efficiency.

Low N availability affected root morphology and photosynthesis in both poplar species (Table 1). Total fine root surface area increased in Pp under both low N levels, but remained unaltered in Pg. Total root volume was unchanged in Pp but decreased in Pg under the lowest N supply level compared to that under the control N supply. Photosynthesis decreased stronger in Pp than those in Pg under N-limiting conditions. PNUE_i_ was lower in both poplar species under limiting N supply levels in comparison with that under the control N condition. As a stored photosynthate, foliar starch concentration was higher in both species under low N levels than those under the control N level. N supply levels had no effects on root starch concentration.

These data show that limiting N levels induces distinct responses of root morphology and photosynthesis in Pp and Pg, which is probably associated with interspecific differences in N metabolism.

### Net fluxes of NH_4_^+^, NO_3_^–^, and H^+^, activities of PM H^+^-ATPases, and accumulation of NH_4_^+^, NO_3_^–^, and NO_2_^–^

**Fig. 2.**
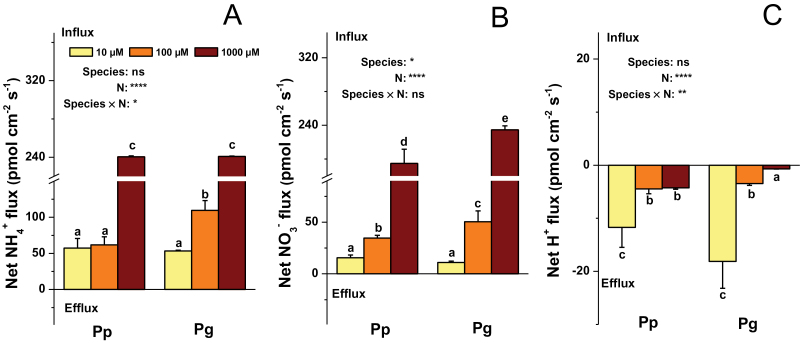
Net fluxes of NH_4_^+^ (A), NO_3_^–^ (B), and H^+^ (C) in 10min at 15mm from the root apex of fine roots of *P. popularis* (Pp) and *P. alba × P. glandulosa* (Pg). Data indicate mean ± SE (*n* = 6). The measuring solution (pH 5.5) contained 0.1mM KCl and 0.1mM CaCl_2_ as well as 10, 100, or 1000 μM NH_4_NO_3_. Bars labelled with different letters indicate significant difference between the treatments. *P*-values of the ANOVAs of species, N treatment, and their interaction are indicated. *P < 0.05; **P < 0.01; ***P < 0.001; ****P < 0.0001; ns, not significant (this figure is available in colour at *JXB* online).

N uptake is the first crucial step, which may lead to distinct differences in N metabolism of Pp and Pg. Net fluxes of NH_4_^+^ and NO_3_^–^ measured along the root tips of Pg displayed large variation at different positions, and maximal net influxes of NH_4_^+^ and NO_3_^–^ occurred at the position of 15mm from the root apex (Supplementary Fig. S1B). Based on these findings in Pg and previous observations in Pp (Luo *et al.*, 2013), net fluxes of NH_4_^+^, NO_3_^–^, and H^+^ were measured with greater detail at the position of 15mm from the root apex of both species (Fig. 2).

**Fig. 3.**
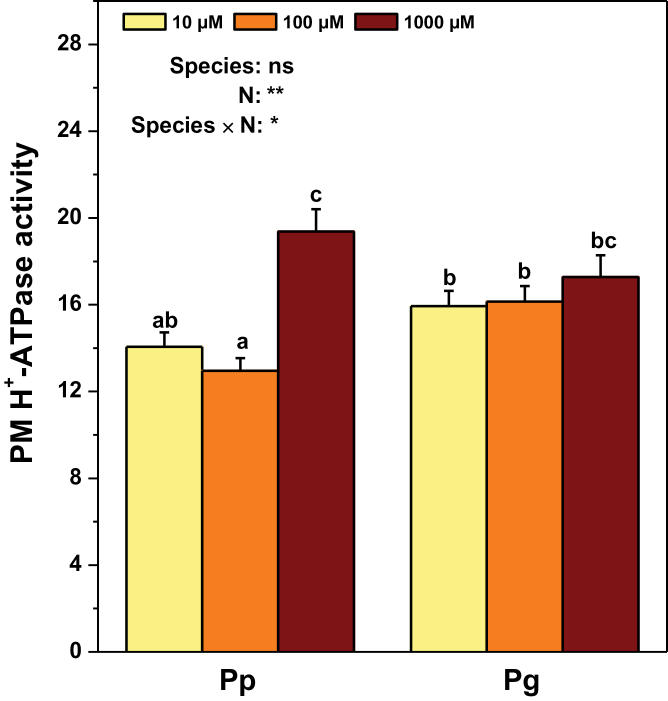
PM H^+^-ATPase activity (mmol Pi h^–1^ (mg protein)^–1^) in roots of *P. popularis* (Pp) and *P. alba × P. glandulosa* (Pg) exposed to 10, 100, or 1000 μM NH_4_NO_3_. Bars indicate mean ± SE (*n* = 6). Different letters on the bars indicate significant difference. *P*-values of the ANOVAs of species, N treatment, and their interaction are indicated. *P < 0.05; **P < 0.01; ***P < 0.001; ****P < 0.0001; ns, not significant (this figure is available in colour at *JXB* online).

Under control N, net NH_4_^+^ influx was similar in roots of Pp and Pg (Fig. 2A), but net NO_3_^–^ influx was higher in roots of Pg than those of Pp (Fig. 2B). Under 100 μM NH_4_NO_3_, Pg exhibited higher net influxes of NH_4_^+^ or NO_3_^–^ than Pp (Fig. 2A, B). Since net influxes of NH_4_^+^ and NO_3_^–^ are associated with H^+^ fluxes (Luo *et al.*, 2013), net H^+^ fluxes were also determined in Pp and Pg under the three N supply levels (Fig. 2C). The net H^+^ efflux was lower in Pg than in Pp under the control N level. Net H^+^ flux at the root surface is coupled with activities of PM H^+^-ATPases (Luo *et al.*, 2013). Thus, PM H^+^-ATPase activities were analysed in isolated plasma membranes of fine roots. The PM H^+^-ATPase activity was similar in Pp and Pg under the control N condition (Fig. 3).

Low N supply levels always resulted in significant decreases in net NO_3_^–^ influxes in Pp and Pg compared to the control N condition (Fig. 2B). Both low N levels led to increased net H^+^ efflux in Pg, but only the lowest N level elevated net H^+^ efflux in Pp in comparison with the control N supply (Fig. 2C). In Pp, the PM H^+^-ATPase activities decreased under low N supply compared to the control N condition, whereas no such effects were found in Pg (Fig. 3).

**Fig. 4.**
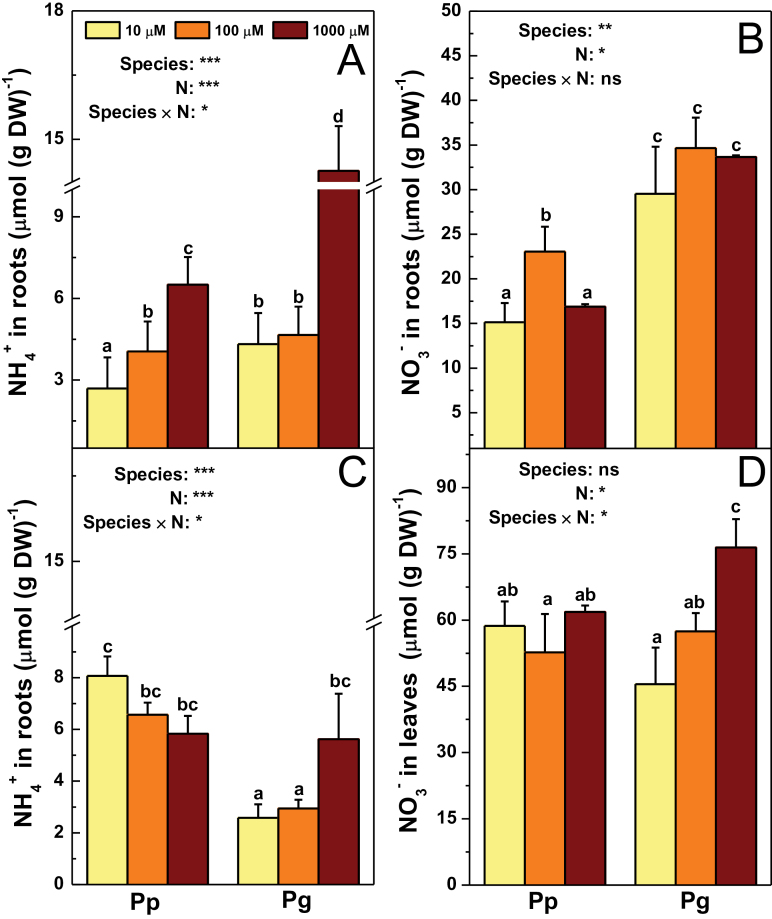
NH_4_^+^ and NO_3_^–^ content in roots (A and B) and leaves (C and D) of *P. popularis* (Pp) and *P. alba × P. glandulosa* (Pg) exposed to 10, 100, or 1000 μM NH_4_NO_3_. Bars indicate mean ± SE (*n* = 6). Different letters on the bars indicate significant difference. *P*-values of the ANOVAs of species, N treatment, and their interaction are indicated. *P < 0.05; **P < 0.01; ***P < 0.001; ****P < 0.0001; ns, not significant. DW, dryweight (this figure is available in colour at *JXB* online).

Different uptake rates of NH_4_^+^ or NO_3_^–^ at the root surface of Pg and Pp exposed to the three N levels may result in differences in NH_4_^+^, NO_3_^–^, and NO_2_^–^ content in plants. Therefore, these compounds were further analysed (Fig. 4 and Supplementary Fig. S3). NH_4_^+^ content was higher in roots of Pg than those of Pp (Fig. 4A). NO_3_^–^ content was higher in roots of Pg than those of Pp under the three N levels (Fig. 4B). Root NO_2_^–^ content was similar in both species (Supplementary Fig. S3). Foliar NH_4_^+^ content was similar in Pp and Pg under the control N supply, but lower in Pg than in Pp under limiting N conditions (Fig. 4C). Foliar NO_3_^–^ content was higher in Pg than that in Pp under the control N level (Fig. 4D).

Low N supply affected NH_4_^+^, NO_3_^–^, and NO_2_^–^ content in poplars. In comparison with the control N level, NH_4_^+^ accumulation decreased in roots of both species in response to low N supply (Fig. 4A). NO_3_^–^ content increased in Pp roots under 100 μM NH_4_NO_3_ compared to that under the control N supply (Fig. 4B). The N treatment had no impacts on root NO_2_^–^ content (Supplementary Fig. S3). Foliar NH_4_^+^ content decreased in Pg in response to low N availability, but remained unaltered in Pp under the three N levels (Fig. 4C). Foliar NO_3_^–^ content decreased in Pg in response to low N supply (Fig. 4D).

### Activities of enzymes involved in N assimilation, total N, δ^15^N, and mineral nutrients

**Fig. 5.**
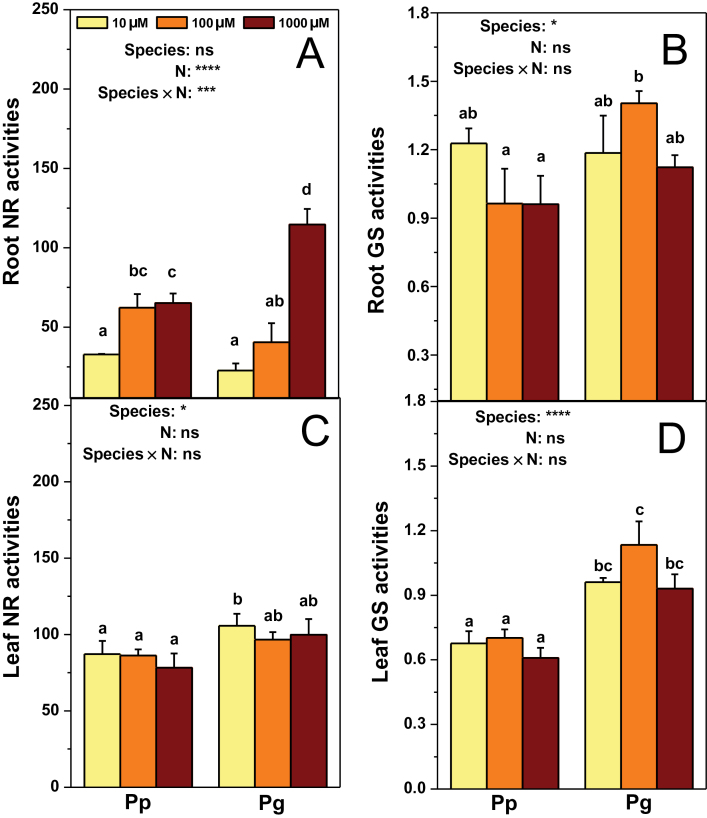
Activities of nitrate reductase (NR, nmol NO_3_^–^ h^–1^ (mg protein)^–1^), and glutamine synthetase (GS, h^–1^ (mg protein)^–1^) in roots (A and B) and leaves (C and D) of *P. popularis* (Pp) and *P. alba × P. glandulosa* (Pg) exposed to 10, 100, or 1000 μM NH_4_NO_3_. Bars indicate mean ± SE (*n* = 6). Different letters on the bars indicate significant difference. *P*-values of the ANOVAs of species, N treatment, and their interaction are indicated. *P < 0.05; **P < 0.01; ***P < 0.001; ****P < 0.0001; ns, not significant (this figure is available in colour at *JXB* online).

After the uptake of NH_4_^+^ and NO_3_^–^, enzymes play important roles in N assimilation. Therefore, this study determined the activities of enzymes (NR, NiR, GS, GOGAT, and GDH) involved in N assimilation in Pp and Pg (Figs. 5 and Supplementary Fig. S4). Root NR activity was higher in Pg than that in Pp under the control N level (Fig. 5A). Root NiR activities were similar in Pp and Pg under the control N supply, but lower in Pg than in Pp under low N supply conditions (Supplementary Fig. S4). Root GS activity was higher in Pg than that in Pp under 100 μM NH_4_NO_3_ (Fig. 5B). Activities of GOGAT or GDH in roots were unaffected by species (Supplementary Fig. S4). In leaves, analysed enzyme activities of Pg were higher than those of Pp (Figs. 5C, D and Supplementary Fig. S4).

Limiting N supply also influenced activities of enzymes implicated in N assimilation in both poplar species. Root NR activity reduced in both species in response to low N levels (Fig. 5A). Root NiR activity was stimulated in Pp by 10 μM NH_4_NO_3_ compared with that under the control N level, but remained unchanged in Pg under the three N levels (Supplementary Fig. S4). Activities of GOGAT or GDH in roots were unaffected by low N levels (Supplementary Fig. S4). In leaves, these enzyme activities in both species remained unaltered in response to low N availability except that foliar GDH activities decreased in Pg and stimulated in Pp exposed to 10 μM NH_4_NO_3_ (Figs. 5C, D and Supplementary Fig. S4).

**Fig. 6.**
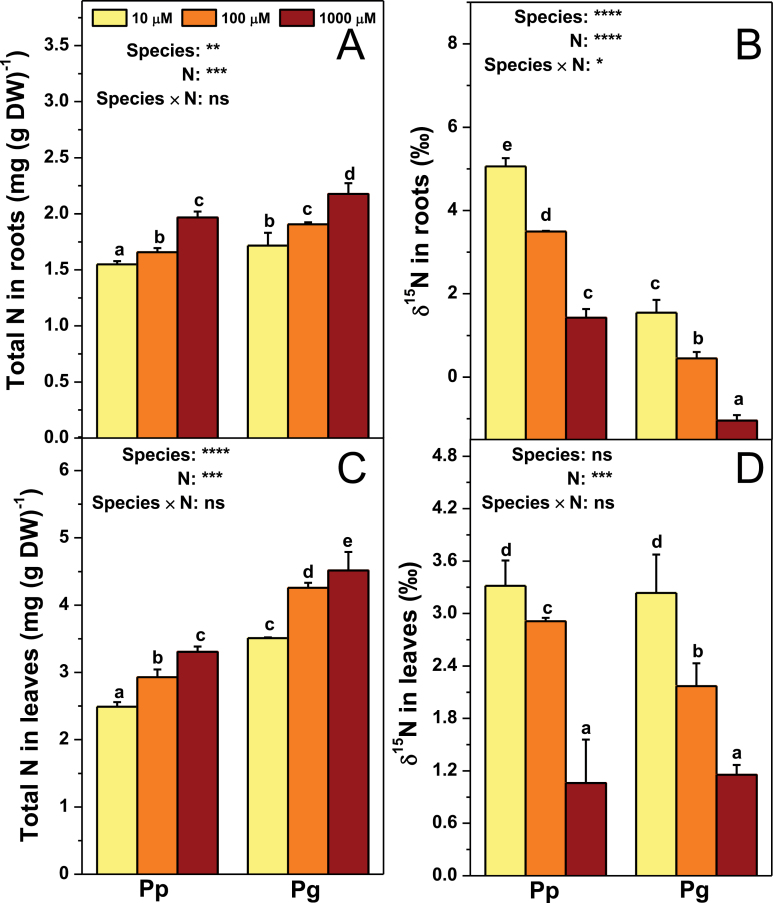
Total N concentration and δ^15^N in roots (A and B) and leaves (C and D) of *P. popularis* (Pp) and *P. alba × P. glandulosa* (Pg) exposed to 10, 100, or 1000 μM NH_4_NO_3_. Bars indicate mean ± SE (*n* = 6). Different letters on the bars indicate significant difference. *P*-values of the ANOVAs of species, N treatment, and their interaction are indicated. *P < 0.05; **P < 0.01; ***P < 0.001; ****P < 0.0001; ns, not significant. DW, dryweight (this figure is available in colour at *JXB* online).

Irrespective of N forms, total N concentration in plants may mirror N availability. Moreover, fractionation of ^15^N may occur in different steps of N metabolism in plants, reflecting active status of N metabolism. Therefore, total N and δ^15^N were analysed in Pp and Pg (Fig. 6). Total N concentration in roots of Pg was higher than those of Pp under the same N level (Fig. 6A). In contrast, δ^15^N in roots of Pg was lower than that of Pp under the same N treatment (Fig. 6B). Foliar N concentration of Pg was also higher than those of Pp under the same N level (Fig. 6C). Foliar δ^15^N was unaffected by species (Fig. 6D).

Total N concentration in roots reduced with decreasing N supply levels in both species (Fig. 6A). On the contrary, δ^15^N in roots of both species increased in response to both low N levels (Fig. 6B). Foliar N concentration in both species was also lower under low N levels compared to those under the control N condition (Fig. 6C). In contrast, foliar δ^15^N in both species increased in response to low N availability (Fig. 6D).

As N availability can also affect uptake of other nutrients and carbon metabolism, mineral nutrients, soluble sugars, total C, δ^13^C, soluble protein, and phenolics were analysed in Pp and Pg (Supplementary Figs. S5 and S6, Supplementary Table S2). There were complex patterns in the responses of nutrient elements and carbon-bearing compounds to N supply levels.

### PCA of morphological and physiological responses

**Fig. 7.**
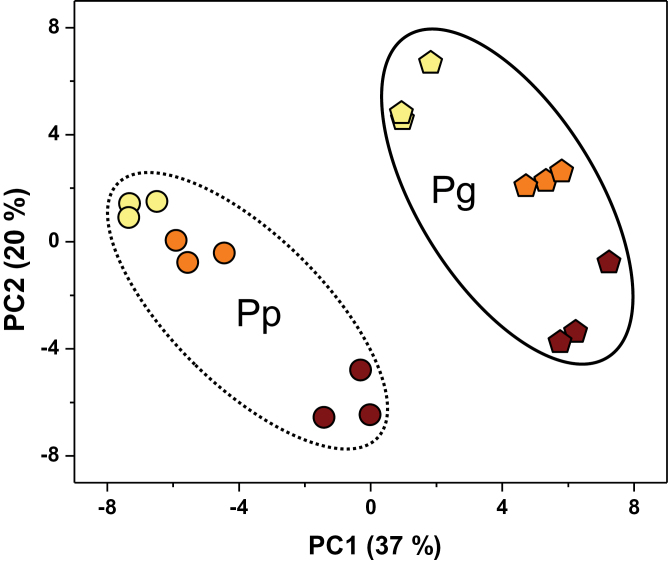
Principal component analysis (PCA) plot of the first two principal components in *P. popularis* (Pp) and *P. alba × P. glandulosa* (Pg). The analysis was conducted using data of physiological parameters of Pp and Pg exposed to 10 (yellow), 100 (orange), or 1000 (brown) μM NH_4_NO_3_, respectively (this figure is available in colour at *JXB* online).

To unravel key parameters involved in the response patterns of both poplar species to N supply levels, a PCA was conducted using data of morphological and physiological parameters related to root morphology, photosynthesis, and N metabolism (Fig. 7, Supplementary Table S3). PC1 and PC2 accounted for 37 and 20% of the variation, respectively. PC1 clearly separated the variation of species effects, and PC2 uncovered the effects of N treatment levels. Foliar N concentration and root δ^15^N were key contributors to PC1, whereas *A*, net influxes of NH_4_^+^ and NO_3_^–^, and foliar starch concentration was important factors to PC2. In the PCA plot, a greater distance between symbols associated with N treatment levels suggests a stronger responsiveness of morphological and physiological parameters to changes in N supply levels. Thus, the greater distance between symbols related to the control N level and 100 μM NH_4_NO_3_ in Pp compared to that in Pg indicates that Pp is more sensitive to decreasing N supply than Pg in the range of given N availability. These PCA results indicate that Pp and Pg exhibit distinct morphological and physiological responsiveness in acclimation to limiting N availability, which mainly results from differences of Pp and Pg in uptake of NH_4_^+^ or NO_3_^–^, root ^15^N fractionation, foliar N and starch concentration, and *A*.

### Transcriptional regulation of genes involved in N metabolism

**Fig. 8.**
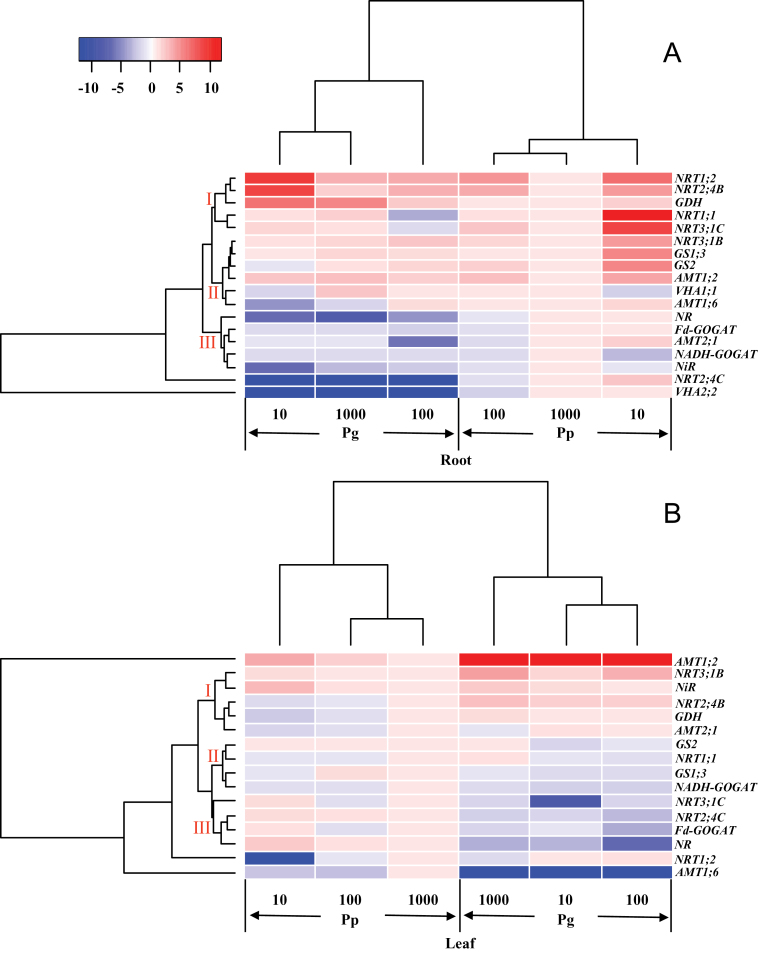
Cluster analysis of transcriptional fold-changes of key genes involved in N uptake and assimilation in roots (A) and leaves (B) of *P. popularis* (Pp) and *P. alba × P. glandulosa* (Pg) exposed to 10, 100, or 1000 μM NH_4_NO_3_. The colour scale indicates fold-changes of mRNAs. For each gene, the expression levels in roots or leaves of Pp exposed to 1000 μM NH_4_NO_3_ were defined as 1, and the corresponding fold-changes under 100 and 10 μM NH_4_NO_3_ were calculated (this figure is available in colour at *JXB* online).

Since Pp and Pg demonstrated distinct patterns of morphological and physiological responses in acclimation to limiting N availability, interspecific differences may also be expected in the transcriptional regulation pattern of key genes implicated in N metabolism. Therefore, transcript levels of representative genes involved in N acquisition and assimilation were assessed in roots and leaves of both species (Fig. 8). The cluster analysis of transcript changes of N uptake- and assimilation-related genes clearly separated Pg and Pp based on their responsiveness to N supply levels (Fig. 8).

In roots, *NRT1;2*, *NRT2;4B*, *GDH*, *NRT1;1*, and *NRT3;1C* formed a subcluster I (Fig. 8A). Under the control N level, the transcript abundance of genes in the subcluster I was higher in Pg than in Pp (Fig. 8A). The second subcluster consisted of *NRT3;1B*, *GS1;3*, *GS2*, *AMT1;2*, *VHA1;1*, and *AMT1;6* and the transcript levels of these genes were similar or lower in Pg compared to those in Pp under the control N level (Fig. 8A). The subcluster III included *NR*, *Fd-GOGAT*, *AMT2;1*, *NADH-GOGAT*, and *NiR*, and the mRNA levels of these genes were lower in Pg than those in Pp under the control N level (Fig. 8A). The strongest differences existed for *NRT2;4C* and *VHA2;2*, which were strongly suppressed in Pg compared to those in Pp under the three N supply levels (Fig. 8A).

Limiting N supply affected transcript levels of genes involved in N metabolism in roots of both species (Fig. 8A). Generally, the transcript levels of genes from the subcluster I were increased in Pp in response to low N levels in comparison with those under the control N condition, but the transcript changes of these genes in Pg were diverse in response to low N availability (Fig. 8A). The transcriptional induction of genes from subcluster II except *VHA 1;1* was detected in Pp in response to 10 μM NH_4_NO_3_ in comparison with those under the control N condition, but no such effects were found in Pg (Fig. 8A). The transcript levels of genes from subcluster III were suppressed in Pp in response to 100 μM NH_4_NO_3_ compared to those under the control N supply, but no such effects were observed in Pg (Fig. 8A). The strongest differences existed for *NRT2;4C* and *VHA2;2* because *NRT2;4C* and *VHA2;2* responded to N supply variation in Pp but not in Pg (Fig. 8A).

In leaves, *NRT3;1B*, *NiR*, *NRT2;4B*, *GDH*, and *AMT2;1* formed a subcluster I (Fig. 8B). Under the control N level, the mRNA levels of genes in the subcluster I except *AMT2;1* were higher in Pg than those in Pp (Fig. 8B). *GS2*, *NRT1;1*, *GS1;3*, and *NADH-GOGAT* constitute the second subcluster (Fig. 8B). The transcript levels of genes from this subcluster were similar or slightly lower in Pg than those in Pp (Fig. 8B). The third subcluster consisted of *NRT3;1C*, *NRT2;4C*, *Fd-GOGAT*, and *NR* (Fig. 8B). Under the control N level, genes from subcluster III showed lower transcript levels in Pg than those in Pp (Fig. 8B). The mRNA level of *AMT1;2* was higher in Pg than that in Pp under the control N condition (Fig. 8B).

In leaves, transcript levels of genes related to N metabolism were also affected by N supply. *NRT2;4B*, *GDH*, and *AMT2;1* from subcluster I had lower transcript levels in Pp in response to low N availability compared to those exposed to the control N supply, but the transcript levels of these genes were relatively stable in Pg under the three N levels (Fig. 8B). In the second subcluster, the mRNA levels of *GS2* were stable in Pp in response to low N availability, but repressed in Pg under low N levels compared to that under the control N condition (Fig. 8B). In comparison with the control N supply, the transcript levels of genes from subcluster III were increased in Pp exposed to 10 μM NH_4_NO_3_, but suppressed or unaltered in Pg (Fig. 8B). The other three genes (i.e. *NRT1;2*, *AMT1;6*, and *AMT1;2*) displayed the strongest differences between Pp and Pg in response to N availability (Fig. 8B). *NRT1;2* and *AMT1;6* showed lower mRNA levels in Pp in response to low N availability, but no such effects were observed in Pg (Fig. 8B). The mRNA level of *AMT1;2* was increased in Pp but not in Pg in response to low N availability (Fig. 8B).

To find out which genes are the most important ones in response to differences in species and/or N supply levels, a PCA was performed using data of fold-changes of transcripts in Pp and Pg under the three N levels (Supplementary Table S4). PC1 clearly separated species effects and PC2 the N treatment impacts. PC1 and PC2 accounted for 48 and 26% of the variation, respectively. Leaf *NRT2;4C* and *AMT1;6*, and root *NRT2;4C* were the most important contributors to PC1, whereas *NADH-GOGAT*, *AMT1;2*, and *NRT1;2* in roots were essential factors in PC2.

## Discussion

### Differences between Pp and Pg in N metabolism under limiting N supply

The greater root biomass and larger fine root surface area of Pp compared with Pg suggest that root morphological features of Pp are more responsive to limiting N availability than those of Pg. More stimulation of root growth in Pp than in Pg can be critical for different acclimation patterns of both species to limiting N availability because N acquisition in these poplars depends on root characteristics. The greater root biomass and fine root surface area in Pp indicate that Pp may better exploit nutrient resources in rhizosphere in comparison with Pg. The higher growth rates of plants may need more N metabolites to support (Lawlor, 2002). Thus, growth can be a driving force for N metabolism of plants. Consequently, the higher root growth of Pp can lead to greater N demand, further triggering a stronger responsiveness to decreasing N availability. Despite lower total N concentration in Pp roots than in Pg roots, the greater root biomass of Pp resulted in higher N amount (14–38%) in Pp roots, suggesting that Pp can acquire more N to support higher root growth.

The greater root growth in Pp, however, does not necessitate higher net influxes of N compared to those in Pg. Actually, Pp displayed lower net influxes of NH_4_^+^ and NO_3_^–^ compared to those of Pg under 100 μM NH_4_NO_3_, which is likely associated with PM H^+^-ATPase activities, activities of enzymes implicated in N assimilation, and functioning of AMTs and NRTs (Hawkins *et al.*, 2008; Hawkins and Robbins, 2010; Alber *et al.*, 2012; Luo *et al.*, 2013). PM H^+^-ATPases play a central role in the uptake of NH_4_^+^ and NO_3_^–^ because H^+^ ions pumping to the apoplast through PM H^+^-ATPases create the proton motive force, driving the absorption of NH_4_^+^ and NO_3_^–^ in roots (Hawkins and Robbins, 2010; Luo *et al.*, 2013). The lower activity of PM H^+^-ATPases is in line with the lower net influxes of NH_4_^+^ and NO_3_^–^ in Pp compared with Pg under 100 μM NH_4_NO_3_. Additionally, in most cases, the lower activities of enzymes involved in N assimilation and the lower total N concentration in roots and leaves of Pp versus Pg correspond well to the lower N uptake rates in Pp under 100 μM NH_4_NO_3_. In contrast to the lower total N concentration in Pp than in Pg, the higher δ^15^N in roots of Pp indicates that ^15^N is more rapidly enriched in the process of N metabolism in roots of Pp than of Pg. Since the processes of N metabolism in plants discriminate against the heavier N isotope leading to the depletion of ^15^N in plant dry mass compared with that in the soil (Tcherkez and Hodges, 2008; Falxa-Raymond *et al.*, 2012; Gauthier *et al.*, 2013), higher δ^15^N in roots of Pp indicates less fractionation of ^15^N occurs in Pp than in Pg. This is consistent with the lower net influxes and content of NH_4_^+^ and NO_3_^–^, activities of NR and GS, and total N concentration in Pp compared with Pg. Based on morphological and physiological parameters related to N metabolism, the PCA results suggest that Pp is more sensitive to decreasing N supply than Pg under 100–1000 μM NH_4_NO_3_, which is mainly due to differences between Pp and Pg in the uptake of NH_4_^+^ or NO_3_^–^, root ^15^N fractionation, foliar N and starch concentration, and *A*.

The distinct patterns of transcriptional regulation of genes implicated in N metabolism of Pp and Pg may be associated with the different morphological and physiological responses of both species to limiting N availability. This study group’s previous study suggests that, under N fertilization, differential expression of genes involved in N uptake (*AMT*s and *NRT*s) of Pp and Pg leads to accelerated N physiological processes in Pg than in Pp (Li *et al.*, 2012). Under limiting N conditions, however, the current data show that transcriptional regulation of key genes involved in N metabolism of Pp is more responsive than that of Pg. These results indicate that Pp and Pg can differentially manage transcriptional regulation of key genes involved in N metabolism under low and high N availability. These results are consistent with previous studies. *Arabidopsis* plants manage N metabolism differently under deficient and sufficient N conditions (Lemaitre *et al.*, 2008; Chardon *et al.*, 2010; Ikram *et al.*, 2012). These studies highlight that it is necessary to investigate the responses of plants not only to high N fertilization but also to low N availability.

Overall, Pp and Pg displayed different patterns of morphological, physiological, and transcriptional regulation in response to limiting N availability, which is mainly associated with the difference of both species in net influxes of NH_4_^+^ and NO_3_^–^, root δ^15^N, foliar N and starch concentration, *A*, and the transcriptional regulation of genes (e.g. *AMT1;2*, *NRT1:2*, and *NRT2;4C* in roots and *AMT1;6* and *NRT2;4C* in leaves) that are involved in N acquisition and assimilation.

### The physiological and transcriptional regulation mechanisms of N metabolism of poplars in acclimation to low N availability

Root morphology responds highly plastic to N availability (Forde and Walch-Liu, 2009; Chapman *et al.*, 2012). Increases in fine root surface area of poplars exposed to low N levels indicate that poplars stimulate growth of fine roots to forage for nutrients under limiting N availability. Consistently, *Arabidopsis* roots adopt an ‘active-foraging strategy’ by outgrowth of lateral roots under limiting N conditions, but a ‘dormant strategy’ by inhibited growth of lateral roots under sufficient N supply (Ruffel *et al.*, 2011). Most plants can increase root growth, resulting in greater fine root surface area under short-term N deficiency, but exhibit stunted root growth under long-term limiting N availability due to lack of internal N (Kraiser *et al.*, 2011; Shen *et al.*, 2013). The current data indicate that poplar roots adopt an active-forage strategy to acquire N resources and other essential minerals under low N supply. Although poplar roots actively forage for nutrients under low N conditions, the acquired N appears insufficient for the biosynthesis of photosynthetic enzymes and metabolic precursors, leading to decreased *A* and PNUE_i_. In other higher plants, N deficiency also inhibits photosynthetic capacity and growth (Sardans and Penuelas, 2012).

Gradual decreases in net influxes of NH_4_^+^ and NO_3_^–^ at the root surface under decreased concentration of external NH_4_NO_3_ supply indicate that NH_4_^+^ and NO_3_^–^ concentration in the external solution play important roles in net influxes of these ions. This was also found for seedlings of Douglas fir (*Pseudotsuga menziesii*) and soybean (*Glycine max*) (Hawkins and Robbins, 2010). However, net influxes of NH_4_^+^/NO_3_^–^ are greater at the root surface of white spruce (*Picea glauca*) exposed to 50 μM NH_4_NO_3_ than those of 1500 μM NH_4_NO_3_ (Alber *et al.*, 2012). These results suggest that impacts of external NH_4_NO_3_ concentration on net fluxes of NH_4_^+^/NO_3_^–^ are also related to plant species. Decreases in PM H^+^-ATPase activities and transcript levels of genes (*VHA1;1* and *VHA2;2*) encoding PM H^+^-ATPases, root NH_4_^+^ and foliar NO_3_^–^ content, foliar GDH activity, total N concentration in roots and leaves, and soluble protein in roots and leaves of both poplar species are in agreement with lower net influxes of NH_4_^+^/NO_3_^–^ under limiting N supply. Moreover, correlations between different enzymes involved in N metabolism, enzymes, gene expression levels, and transcript levels of different genes in both poplar species (Supplementary Fig. S7) indicate that poplar plants coordinate each step of N metabolism processes to acclimate to low N availability. These results indicate that the processes of N uptake and assimilation have slowed down in both poplar species in acclimation to low N availability. In plants, N and C metabolism is interconnected because the production of N metabolites such as amino acids needs C skeletons (Nunes-Nesi *et al.*, 2010). The lower total C concentration in roots and foliar δ^13^C, the elevated starch concentration in leaves, and the correlations between δ^13^C and parameters of N metabolism in both poplar species under low N levels (Supplementary Figs. S5 and S7) suggest that C export and/or phloem transport is inhibited under N deficiency. Foliar starch accumulation is also observed in herbaceous plants in response to low N availability, which is ascribed to the reduced demand of C skeletons for N compounds such as amino acids and proteins under low N levels (Lemaitre *et al.*, 2008; Ikram *et al.*, 2012; Schluter *et al.*, 2012). In the same line, earlier studies indicate that foliar starch accumulation is a consequence of photosynthesis exceeding the demands of respiration and growth under N deficiency conditions (Lawlor *et al.*, 1987b; Lawlor, 2002).

Increases in δ^15^N in roots and leaves of poplars under low N levels are contrary to decreases in total N concentration, indicating that ^15^N is enriched in both poplar species under limiting N availability. N starvation also stimulates whole-plant δ^15^N in some genotypes of wild barley (*Hordeum spontaneum*) (Robinson *et al.*, 2000). The higher δ^15^N values in both poplar species under low N conditions compared with that under the control N supply are probably associated with slowing down of N metabolism which leads to less depletion of ^15^N in poplars under low N availability. In other words, poplar plants are forced to utilize ^15^N to meet their N demands under limiting N conditions, leading to ^15^N enrichment in dry mass. Although the mechanisms underlying the variation in natural ^15^N are not completely known in plants under various environmental conditions (Cernusak *et al.*, 2009; Tcherkez, 2011), ^14^N/^15^N fractionation can occur in the processes of N absorption, assimilation, recycling, and reallocation in plants and N release from plants (e.g. foliar NH_3_ volatiles and N-containing exudates of roots; Robinson *et al.*, 2000; Ariz *et al.*, 2011; Gauthier *et al.*, 2013; Yousfi *et al.*, 2013). Additionally, changes in environmental factors such as nutrients, can cause substantial alterations in δ^15^N in plants (Ariz *et al.*, 2011; Gauthier *et al.*, 2013; Yousfi *et al.*, 2013). Furthermore, negative correlations between δ^15^N and transpiration rate were observed in wheat under salinity (Yousfi *et al.*, 2013). Consistently, negative correlations occur between δ^15^N and transpiration rate, *g*_s_, *A*, or PNUE_i_ in poplars (Supplementary Fig. S7). These results indicate that ^15^N enrichment in poplars exposed to low N levels is probably associated with (i) less active N metabolism in poplars under low N availability and/or (ii) elevated N release from roots and leaves. The latter possibility needs further studies.

Although transcriptional regulation of genes involved in N metabolism plays a fundamental role in response to N deficiency or starvation in herbaceous plants (Hirai *et al.*, 2004; Bi *et al.*, 2007; Krouk *et al.*, 2010; Patterson *et al.*, 2010; Krapp *et al.*, 2011; Ruffel *et al.*, 2011; Engelsberger and Schulze, 2012; Kiba *et al.*, 2012; Schluter *et al.*, 2012), little is known on transcriptional regulation underlying N metabolism in trees under limiting N availability (Rennenberg *et al.*, 2009, 2010). Transcriptional induction of several *AMTs* (e.g. *AMT1;2*) and *NRTs* (e.g. *NRT1;2*, *NRT2;4B*, and *NRT3;1B*) in poplar roots exposed to low N levels indicates that poplar roots increase mRNAs of key transporters for NH_4_^+^ and NO_3_^–^ as the result of acclimation to low N availability. Induced transcript abundance of *AMT1;2* is also found in roots of *P. tremula* × *tremuloides* exposed to low N supply (Selle *et al.*, 2005) and in *P. tremula* × *alba* under N starvation (Couturier *et al.*, 2007). Similarly, transcription of *OsAMT1;2* in roots of rice (*Oryza sativa*) is induced by NH_4_^+^ deficiency (Sperandio *et al.*, 2011). *NRT2;1* (i.e., *NRT2;4C* re-defined in this study) displays higher transcript levels in NO_3_^–^-fed *P.* × *canescens* roots than in NH_4_^+^-fed roots (Ehlting *et al.*, 2007) and is induced upon application of NO_3_^–^ to N-deprived roots of peach (*Prunus persica*) seedlings (Nakamura *et al.*, 2007). NRT3;1 (also called NAR2;1) is a key player in a two-component system including NRT2s for nitrate transport in *Arabidopsis* (Yong *et al.*, 2010; Kotur *et al.*, 2012) and rice (Yan *et al.*, 2011). Correlation analysis between transcript levels of *NRT3;1B* or *NRT3;1C* and other *NRTs* (i.e., *NRT1;1*, *NRT2;4B*, and *NRT2;4C*) in Pp and Pg under normal and low N levels detected positive relationships (Supplementary Fig. S7). These correlations, combined with this study group’s previous findings where positive correlations also occurred under N-fertilizaiton conditions (Li *et al.*, 2012), indicate that NRT3;1B and/or NRT3;1C may also act as partners of other NRTs for nitrate transport in poplars under various N levels. In contrast to induction of several *AMTs* and *NRTs* in poplar roots, reduced transcript levels of most *AMTs* and *NRTs* in leaves and genes (e.g. *NR*, *NiR*, *GOGAT*) involved in N assimilation in roots and leaves of poplars indicate that N assimilation (downstream processes after N uptake) is inhibited due to shortage of N-containing precursors under low N availability. These results suggest that overexpression of *AMTs* and *NRTs* in poplar roots and downregulation of most *AMTs* and *NRTs* in leaves and genes involved in N assimilation in roots and leaves of poplars play fundamental roles in acclimation to limiting N availability.

Taken together, increased fine root growth, slowed down N acquisition and assimilation, overexpressed transcripts of *AMTs* and *NRTs* in roots, and repressed transcript levels of *AMTs* and *NRTs* in leaves and key genes involved in N assimilation are primary mechanisms of both poplar species in acclimation to limiting N availability.

In summary, Pp exhibited greater root biomass and total fine root surface area, lower net influxes of NO_3_^–^ at the root surface, higher δ^15^N in roots, and more responsiveness of transcriptional regulation of 18 genes involved in N uptake and assimilation in roots and leaves than Pg under limiting N supply. These results indicate that N metabolism of Pp displays a stronger responsiveness to decreasing N availability than that of Pg. Under low N conditions, decreased net influxes of NH_4_^+^ and NO_3_^–^ at the root surface are consistent with lower root NH_4_^+^ and foliar NO_3_^–^ content, root NR activity, total N concentration in roots and leaves, and mRNA of most *AMTs* and *NRTs* in leaves and genes involved in N assimilation in roots and leaves. Moreover, low N supply levels increased fine root surface area, foliar starch accumulation, δ^15^N in roots and leaves, and transcript levels of several *AMTs* (e.g. *AMT1;2*) and *NRTs* (e.g. *NRT1;2*, *NRT2;4B*, and *NRT3;1B*) in roots of both poplar species. These data suggest that poplar species slow down processes of N acquisition and assimilation in acclimation to limiting N supply. These morphological, physiological, and molecular data suggest that poplar plants can differentially manage N metabolism under deficient and sufficient N conditions and that it is important to consider low N tolerance when selecting woody plants such as *Populus* spp. for energy plantations on nutrient-poor sites. Using technologies including genomics, transcriptomics (e.g. microarray, RNA sequencing). and metabolomics in future experiments, a deeper understanding of poplars in acclimation to low N availability may be obtained.

## Supplementary material

Supplementary data are available at *JXB* online.

Supplementary Table S1. Primers used for qRT-PCR.

Supplementary Table S2. Concentrations of mineral nutrients.

Supplementary Table S3. PCA of physiological parameters of both poplar species.

Supplementary Table S4. PCA of transcriptional changes of representative genes.

Supplementary Fig. S1. Net fluxes of NH_4_^+^ and NO_3_^–^ along the root tip.

Supplementary Fig. S2. Alignments of representative genes.

Supplementary Fig. S3. NO_2_^–^ content in roots and leaves.

Supplementary Fig. S4. Activities of NiR, GOGAT, and GDH in roots and leaves.

Supplementary Fig. S5. Soluble sugars, C concentration, and δ^13^C.

Supplementary Fig. S6. Soluble protein and phenolics.

Supplementary Fig. S7. Correlations of related parameters.

Supplementary Data
