# Supplementary material for: Nitrogen metabolism of two contrasting poplar species during acclimation to limiting nitrogen availability
Source: J Exp Bot. 2013 Aug 20;64(14):4207–24. doi: 10.1093/jxb/ert234 (PMC3808312; doi:10.1093/jxb/ert234)
Supplement: Supplementary Data [file supp_64_14_4207__index.html]

Nitrogen metabolism of two contrasting poplar species during acclimation to limiting nitrogen availability — Nitrogen metabolism of two contrasting poplar species during acclimation to limiting nitrogen availability — Supplementary Data 

# Nitrogen metabolism of two contrasting poplar species during acclimation to limiting nitrogen availability

## 

Data files

**Files in this Data Supplement:**

- Supplementary Data - Supplementary Data
- Supplementary Data - Supplementary Data
