# Supplementary material for: Nitrogen metabolism of two contrasting poplar species during acclimation to limiting nitrogen availability
Source: J Exp Bot. 2013 Aug 20;64(14):4207–24. doi: 10.1093/jxb/ert234 (PMC3808312; doi:10.1093/jxb/ert234)
Supplement: Supplementary Data [file supp_ert234_jexbot100867_file002.pdf]

Supplementary materials to:

Nitrogen metabolism of two contrasting poplar species in acclimation to limiting nitrogen availability

Jie Luo, Hong Li, Tongxian Liu, Andrea Polle, Changhui Peng, Zhi-Bin Luo*

*Correspondence: **Zhi-Bin Luo**
E-mail: luozbbill@163.com

Supplementary Table S1. Primers used for qRT-PCR.

Gene model	Accession number	Gene Name	Closest AGI	Primer-Forward	Primer-Reverse	PCR efficiency (%)
a		*AMT1;2*	AT4G13510	5'-GATGGCATTATAAAGGCATCAAA-3'	5'-GAAGAAGGCGTACGTGAATGA-3'	101
Potri.009G045200 [b]	KC256927[Pp], KC256928[Pg]	*AMT1;6*	AT1G64780	5'-TTCAGGTGAGGCGGGAGT-3'	5'-CCAAAGCGCCAATGATACAG-3'	104
Potri.006G102800 [b]	KC256925[Pp], KC256926[Pg]	*AMT2;1*	AT2G38290	5'-GTTATAGGTTGGAACGTGGTGT-3'	5'-TCAATATTACATGCGTCAAGATTC-3'	104
Potri.003G111500 [b]	KC256933[Pp], KC256934[Pg]	*NRT1;1*	AT1G12110	5'-CTAAACCAAGGGAGGCTCCATGAT-3'	5'-CCCAACACAAAAGTAGGCGAAAAG-3'	100
Potri.012G070700 [b]	KC256935[Pp], KC256936[Pg]	*NRT1;2*	AT1G69850	5'-TCTTTGGTAGCAACTTGAACAA-3'	5'-TCTCTCTCTCTCTCGTCTCCCT-3'	100
Potri.009G008500 [b]	KC256937[Pp], KC256938[Pg]	*NRT2;4B*	AT5G60770	5'-AATAGAGGAAGGGAATGGCTG-3'	5'-TGAGGTTGTCCCGAATGATAG-3'	98
Potri.009G008600 [b]	KC256939[Pp], KC256940[Pg]	*NRT2;4C*	AT1G08090	5'-CAGTCCCGACAGATACAAC-3'	5'-CTTCCCACTACAACGATTTC-3'	98
Potri.015G085000 [b]		*NRT3;1B*	AT5G50200	5'-TCATAGCCTCTTCTTCTACCTTTCC-3'	5'-CCACCTTTCAATACTTGTCCG-3'	105
Potri.015G085100 [b]		*NRT3;1C*	AT5G50200	5'-AGAGGTCTCAGTGAAGCGAACAAG-3'	5'-CGCAAATACAAACGCAATTATCAT-3'	105
Potri.005G172400 [c]	KC256931[Pp], KC256932[Pg]	*NR*	AT1G37130	5'-ATCATCGGATCGGAGAGTTGG-3'	5'-GACGGT-GCTAGTTGGCGTATAG-3'	99
Potri.004G140800 [d]		*NiR*	AT2G15620	5'-ACAAGTTGCCGATATTGGGTTCAT-3'	5'-CCTCTATCACCCGTCGTAGTCCTG-3'	98
Potri.017G131100 [c]	DQ855559	*GS1;3*	AT5G37600	5'-GGCGCAGACCAAGCTTCTC-3'	5'-GCGAAGTGACAGATTTAGGATTGC-3'	100
Potri.010G029100 [c]	DQ855560	*GS2*	AT5G35630	5'-ATCAGGTGGGTCCCAGTGTG-3'	5'-GCGAAGTGACAGATTTAGGATTGC-3'	95
Potri.016G036900 [c]	DQ855561	*Fd-GOGAT*	AT5G04140	5'-AACCCAAAGGCATCAGACTCAG-3'	5'-AGTAAAGCAGGTCCATCCCAAG-3'	101
Potri.012G011700 [c]	KC256929[Pp], KC256930[Pg]	*NADH-GOGAT*	AT5G53460	5'-GGTGTTGTGGATATTCCTCCTG-3'	5'-TCAGATGCGGCGACAACCC-3'	95
Potri.013G058300 [d]		*GDH*	AT5G18170	5'-ATTTATGCTAACTCAGGAGGCGTT-3'	5'-GAGGAGGAAACTAGGGCAATACAT-3'	102
Potri.018G006000 [d]		*VHA1;1*	AT2G24520	5'-CCGCTCAGACCAACACTATCTT-3'	5'-TCCTCTGGGCTTGAATGGTAG-3'	96
Potri.018G090300 [d]		*VHA2;2*	AT2G18960	5'-GGCTCCAACCACCTGAGACAG -3'	5'-AGCACAGCCCTTTCTCTTCCA-3'	104
e		*Actin 2/7*		5'-CCCATTGAGCACGGTATTGT-3'	5'-TACGACCACTGGCATACAGG-3'	101

a: The gene name was from Selle A et al (2005) The high affinity poplar ammonium importer PttAMT1.2 and its role in ectomycorrhizal symbiosis. New Phytologist 168, 697–706. However, we re-designed primers for qRT-PCR;
b: From Li H et al (2012) N-fertilization has different effects on the growth, carbon and nitrogen physiology, and wood properties of slow- and fast-growing *Populus* species. Journal of Experimental Botany 63, 6173-6185;
c: From Dluzniewska P et al (2007) Nitrogen uptake and metabolism in *Populus* × *canescens* as affected by salinity. New Phytologist 173, 279-293;
d: These primers were designed by ourselves based on the database of phytozome *Populus trichocarpa* v3.0;
e: From Brunner AM et al (2004) Validating internal controls for quantitative plant gene expression studies. BMC Plant Biology 4:14;
Pp: Genes cloned from *P. popularis*; Pp: Genes cloned from *P. alba* × *P. glandulosa*.

Supplementary Table S2. Mineral nutrients (μmol g^{-1} DW) in roots and leaves of *P. popularis* (Pp) and *P. alba* × *P. glandulosa* (Pg) exposed to 10, 100 or 1000 μM NH_4NO_3. Data indicate means ± SE (n = 6). Different letters behind the values in the same column indicate significant difference. *P*-values of the ANOVAs of species, N treatment (N) and their interaction are indicated. *: *P*<0.05; **: *P*<0.01; ***: *P*<0.001; ****: *P*<0.0001; ns: not significant.

Species	Tissues	N treatment (μM）	P	S	Na	K	Ca	Mg	Mn	Fe	Al
Pp	Roots	10	506.9 ±82.8 a	102.1 ±0.64 d	61.8 ±9.6 a	333.9 ±2.9 c	1545.9 ±232.1 b	342.0 ±16.6 d	6.68 ±0.8 b	103.7 ±8.6 b	136.6 ±7.2 c
	Roots	100	730.0 ±72.9 b	78.6 ±0.66 ab	63.2 ±4.2 a	266.2 ±5.6 b	1330.2 ±134.6 ab	338.1 ±18.6 c	7.8 ±0.9 b	175.2 ±4.9 c	146.8 ±6.4 c
	Roots	1000	645.2 ±64.6 ab	82.4 ±3.38 bc	72.0 ±8.1 a	171.5 ±16.0 a	1302.1 ±14.7 ab	251.8 ±4.4 b	6.8 ±0.7 b	87.5 ±1.8 b	143.4 ±5.0 c
Pg	Roots	10	715.1 ±52.3 b	87.8 ±5.55 c	82.5 ±8.1 a	345.8 ±5.7 d	1559.4 ±187.5 b	290.5 ±3.8 c	4.3 ±0.4 a	43.9 ±6.2 a	48.6 ±6.7 a
	Roots	100	697.6 ±66.1 ab	79.0 ±1.3 abc	70.2 ±0.8 a	342.8 ±8.2 d	1041.0 ±106.6 a	191.9 ±11.1 a	3.8 ±0.3 a	33.4 ±1.9 a	50.6 ±2.5 a
	Roots	1000	693.8 ±56.6 ab	72.6 ±2.0 a	74.8 ±8.1 a	259.2 ±10.3 b	1063.2 ±81.3 a	167.4 ±0.42 a	3.8 ±0.1 a	35.8 ±5.8 a	80.1 ±5.4 b
Pp	Leaves	10	100.2 ±4.6 a	70.9 ±1.2 a	20.93±1.0 a	460.2 ±0.9 a	254.6 ±14.0 cd	164.4 ±8.9 ab	0.8 ±0.1 bc	1.8 ±0.1 a	3.6 ±0.4 a
	Leaves	100	112.0 ±6.8 ab	79.6 ±2.0 ab	21.5 ±0.9 a	508.3 ±19.6 ab	256.1 ±5.6 cd	178.0 ±7.4 b	0.9 ±0.2 c	2.3 ±0.3 ab	4.0 ±0.7 a
	Leaves	1000	112.1 ±5.1 ab	73.7 ±3.9 ab	23.0 ±1.3 a	504.6 ±13.3 ab	157.9 ±6.5 a	140.6 ±4.4 a	0.6 ±0.1 ab	2.1 ±0.1 a	5.1 ±0.9 ab
Pg	Leaves	10	109.6 ±6.8 ab	93.3 ±4.8 bc	19.7 ±1.2 a	508.3 ±33.2 ab	277.2 ±7.9 d	184.6 ±17.9 b	0.5 ±0.2a	2.6 ±0.1 b	8.7 ±0.3 c
	Leaves	100	123.8 ±11.8 b	114.9 ±13.5 c	25.3 ±2.0 a	621.2 ±42.0 c	231.7 ±5.7 c	181.2 ±15.5 b	0.4 ±0.1 a	2.0 ±0.1 a	6.3 ±0.6 bc
	Leaves	1000	124.8 ±8.4 b	107.1 ±9.2 c	22.8 ±5.4 a	579.3 ±44.8 bc	200.2 ±11.7 b	155.6 ±12.3 ab	0.6 ±0.1 ab	2.6 ±0.2 b	7.5 ±0.3 bc
P-Values	Roots	Species	ns	**	ns	****	ns	****	****	****	****
	Roots	N	ns	***	ns	****	*	****	ns	****	*
	Roots	Species × N	ns	ns	ns	**	ns	*	ns	****	*
	Leaves	Species	ns	***	ns	**	ns	ns	***	*	****
	Leaves	N	ns	ns	ns	*	****	*	ns	ns	ns
	Leaves	Species × N	ns	ns	ns	ns	**	ns	**	**	*

Supplementary Table S3. PCA of physiological parameters of both poplar species exposed to 10, 100 and 1000 μM NH_4NO_3.

Variables	PC1	PC2	PC3	PC4	PC5	PC6
Root biomass	-0.137	-0.020	-0.124	-0.235	-0.139	0.012
Primary root length	-0.107	0.111	-0.155	0.060	0.018	-0.063
Total root length	-0.108	-0.027	-0.166	0.277	0.052	-0.061
Fine root root length	-0.155	-0.059	-0.217	0.057	-0.016	0.036
Fine root to total root length ratio	-0.157	0.039	-0.125	0.101	0.012	0.082
Root average diameter	0.176	0.024	0.062	-0.086	0.066	-0.060
Root surface area	0.015	-0.146	-0.298	0.079	0.132	0.031
Fine root surface area	-0.158	0.028	-0.116	0.153	0.008	0.083
Root volume	0.165	-0.027	-0.051	-0.053	0.100	-0.126
Leaf dry weight	0.018	-0.066	-0.013	0.118	-0.097	0.307
Specific leaf area	0.117	0.084	-0.002	0.136	0.061	-0.351
Net photosynthetic rate (*A*)	0.082	-0.226	0.013	0.055	-0.119	0.006
Stomatal conductance (g_s)	0.171	-0.088	0.065	0.051	-0.048	0.035
Transpiration rate (*E*)	0.167	-0.085	0.082	0.070	-0.092	0.038
Ratio of intercellular CO_2 to ambient CO_2 concentration (C_i/C_a)	0.153	0.050	0.042	0.080	-0.092	0.026
Intrinsic water use efficiency (WUE_i)	-0.146	-0.015	-0.027	-0.102	0.167	-0.054
Instantaneous photosynthetic N use efficiency ($PNUE_i$)	0.085	-0.161	0.036	0.160	-0.087	-0.252
NH_4^+ fluxes	0.102	-0.213	0.063	-0.014	-0.032	0.044
NO_3^- fluxes	0.098	-0.212	0.032	0.028	0.014	0.070
H^+ fluxes	0.064	-0.158	-0.223	-0.175	-0.086	0.122
PM H^+-ATPase	0.096	-0.137	0.223	0.085	-0.030	-0.121
Root Ammonioum	0.137	-0.139	-0.001	0.104	0.085	0.106
Root Nitrate	0.142	0.076	-0.104	0.073	0.141	0.020
Root Nitrite	0.042	-0.013	-0.015	-0.189	0.421	0.088
Leaf Ammonioum	-0.116	-0.117	-0.024	-0.087	0.187	0.012
Leaf Nitrate	0.057	-0.149	0.014	0.011	0.327	-0.098
Leaf Nitrite	-0.063	0.199	0.209	0.107	-0.015	0.061
Root nitrate reductase (NR)	0.074	-0.174	-0.116	0.023	0.129	0.127
Root nitrite reductase (NiR)	-0.135	0.031	0.021	-0.014	0.129	-0.051
Root glutamine synthetase (GS)	0.053	0.136	-0.010	-0.309	-0.075	0.019
Root glutamate synthase (GOGAT)	-0.054	0.085	0.059	-0.026	0.149	-0.312
Root glutamate dehydrogenease (GDH)	-0.047	-0.038	0.082	0.170	0.086	-0.281
Leaf nitrate reductase (NR)	0.081	0.147	-0.007	-0.020	-0.134	0.132
Leaf nitrite reductase (NiR)	0.161	0.061	0.019	0.015	-0.130	-0.072
Leaf glutamine synthetase (GS)	0.140	0.144	-0.068	-0.033	-0.012	-0.107
Leaf glutamate synthase (GOGAT)	0.072	0.115	-0.103	-0.248	0.097	-0.022
Leaf glutamate dehydrogenease (GDH)	0.120	0.011	-0.004	-0.103	0.212	0.267
Root N concentrations	0.151	-0.117	-0.046	-0.001	-0.035	0.056

Continued:

Root $\delta^{15}N$	-0.186	0.052	0.013	-0.058	0.010	-0.058
Root protein	0.136	0.029	-0.064	0.130	0.098	0.223
Leaf N concentrations	0.187	-0.003	-0.075	-0.003	0.001	-0.032
Leaf $\delta^{15}N$	-0.109	0.182	-0.007	0.012	0.002	-0.005
Leaf protein	-0.090	-0.192	-0.001	0.007	0.087	-0.127
Root P	0.066	0.021	-0.189	0.281	-0.043	-0.161
Root S	-0.121	0.091	0.169	-0.121	0.004	0.053
Root Na	0.079	0.063	0.093	0.014	-0.142	0.084
Root K	0.005	0.242	-0.011	-0.094	0.136	-0.045
Root Ca	-0.102	0.079	0.154	0.074	-0.175	0.050
Root Mg	-0.180	0.064	0.017	0.097	-0.041	0.003
Root Mn	-0.153	-0.084	-0.056	-0.004	-0.168	-0.003
Root Fe	-0.159	-0.052	-0.198	0.063	-0.053	0.034
Root Al	-0.146	-0.153	-0.074	-0.022	-0.058	0.045
Leaf P	0.109	-0.008	-0.158	-0.087	-0.202	-0.031
Leaf S	0.151	0.082	-0.155	-0.012	-0.055	-0.109
Leaf Na	0.056	-0.032	-0.130	-0.025	-0.071	-0.101
Leaf K	0.135	0.024	-0.191	-0.063	-0.083	-0.176
Leaf Ca	-0.067	0.218	-0.137	0.067	0.025	0.049
Leaf Mg	0.004	0.185	-0.207	0.021	-0.170	-0.019
Leaf Mn	-0.140	-0.067	-0.168	0.094	0.134	0.097
Leaf Fe	0.066	0.032	-0.036	0.326	0.024	0.160
Leaf Al	0.136	0.084	0.145	0.171	0.028	0.133
Root C concentrations	0.173	-0.079	-0.079	-0.021	0.040	0.026
Root $\delta^{13}C$	-0.138	-0.135	0.067	-0.111	-0.075	0.104
Root soluble sugar	0.116	0.079	-0.064	0.005	0.227	0.076
Root starch	-0.025	0.136	-0.220	0.200	0.043	0.029
Root phenolics	0.102	0.185	0.074	0.096	0.148	0.031
Leaf C concentrations	-0.096	0.035	0.251	0.133	0.056	0.143
Leaf $\delta^{13}C$	-0.088	-0.135	0.168	0.123	0.034	-0.002
Leaf soluble sugar	-0.129	-0.182	-0.028	-0.017	0.059	-0.041
Leaf starch	0.003	0.204	0.031	0.126	0.146	0.110
Leaf phenolics	-0.138	-0.129	0.118	0.032	0.094	-0.140
Proportion of variation (%)	37	20	7	6	5	4

Supplementary Table S4. PCA of transcriptional changes of representative genes involved in N uptake and assimilation in roots (A) and leaves (B) of *P. popularis* (Pp) and *P. alba* × *P. glandulosa* (Pg) exposed to 10, 100 or 1000 μM NH_4NO_3. The PCA plot was also shown below. In PCA plot, data points are presented for 10 (yellow), 100 (orange) and 1000 (wine) μM NH_4NO_3.

Variables	PC1	PC2	PC3
Root *AMT1;2*	-0.085	0.296	-0.095
Leaf *AMT1;2*	0.221	0.091	-0.075
Root *AMT1;6*	-0.169	-0.097	-0.272
Leaf *AMT1;6*	-0.227	-0.126	0.024
Root *AMT2;1*	-0.169	0.045	0.249
Leaf *AMT2;1*	0.154	-0.131	0.164
Root *NRT1;1*	-0.179	0.205	0.052
Leaf *NRT1;1*	-0.001	-0.175	-0.024
Root *NRT1;2*	0.057	0.285	0.218
Leaf *NRT1;2*	0.178	-0.215	0.076
Root *NRT2;4B*	0.098	0.230	0.272
Leaf *NRT2;4B*	0.216	-0.071	-0.057
Root *NRT2;4C*	-0.223	-0.096	-0.060
Leaf *NRT2;4C*	-0.227	-0.024	0.166
Root *NRT3;1B*	-0.130	0.224	-0.215
Leaf *NRT3;1B*	0.151	0.050	-0.355
Root *NRT3;1C*	-0.185	0.211	0.075
Leaf *NRT3;1C*	-0.196	-0.103	-0.251
Root *NR*	-0.219	-0.072	0.084
Leaf *NR*	-0.220	-0.006	0.194
Root *NiR*	-0.185	-0.194	-0.147
Leaf *NiR*	-0.094	0.257	-0.116
Root *GS1;3*	-0.154	0.226	-0.131
Leaf *GS1;3*	-0.121	-0.218	0.127
Root *GS2*	-0.194	0.117	-0.247
Leaf *GS2*	-0.190	-0.083	-0.129
Root *Fd-GOGAT*	-0.208	-0.021	0.146
Leaf *Fd-GOGAT*	-0.193	0.009	0.249
Root *NADH-GOGAT*	0.021	-0.305	0.156
Leaf *NADH-GOGAT*	-0.139	-0.231	0.159
Root *GDH*	0.173	0.166	0.115
Leaf *GDH*	0.202	-0.140	-0.002
Root *VHA1;1*	0.058	-0.225	-0.280
Root *VHA2;2*	-0.221	-0.106	-0.036
Proportion of variation (%)	48	26	16

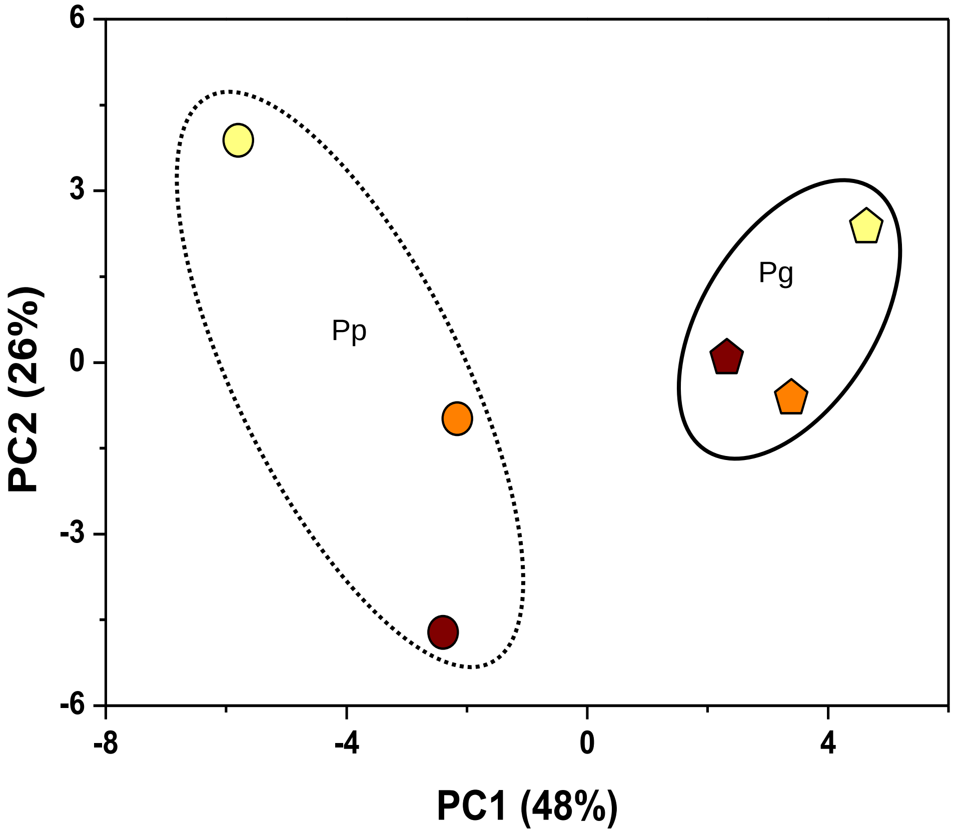

PC2 (26%)
PC1 (48%)
6
3
0
-3
-6
-8
-4
0
4
Pp
Pg

Supplementary Fig. S1. Root tip (a), net fluxes of NH_4^+ (b) and NO_3^- (c) along the root tips of fine roots of *P. alba* × *P. glandulosa* (Pg) exposed to 100 μM NH_4NO_3. Data indicate means ± SE (n = 6). The measuring solution contained 0.1 mM KCl and 0.1 mM $CaCl_2$, pH 5.5, besides either 0.1 mM NH_4Cl for NH_4^+ or 0.1 mM KNO_3 for NO_3^-. For net fluxes of NH_4^+ and NO_3^- along root tips of fine roots of *P. popularis* (Pp), please see Luo et al (2013) Net fluxes of ammonium and nitrate in association with H^+ fluxes in fine roots of *Populus popularis*. Planta 237: 919-931.

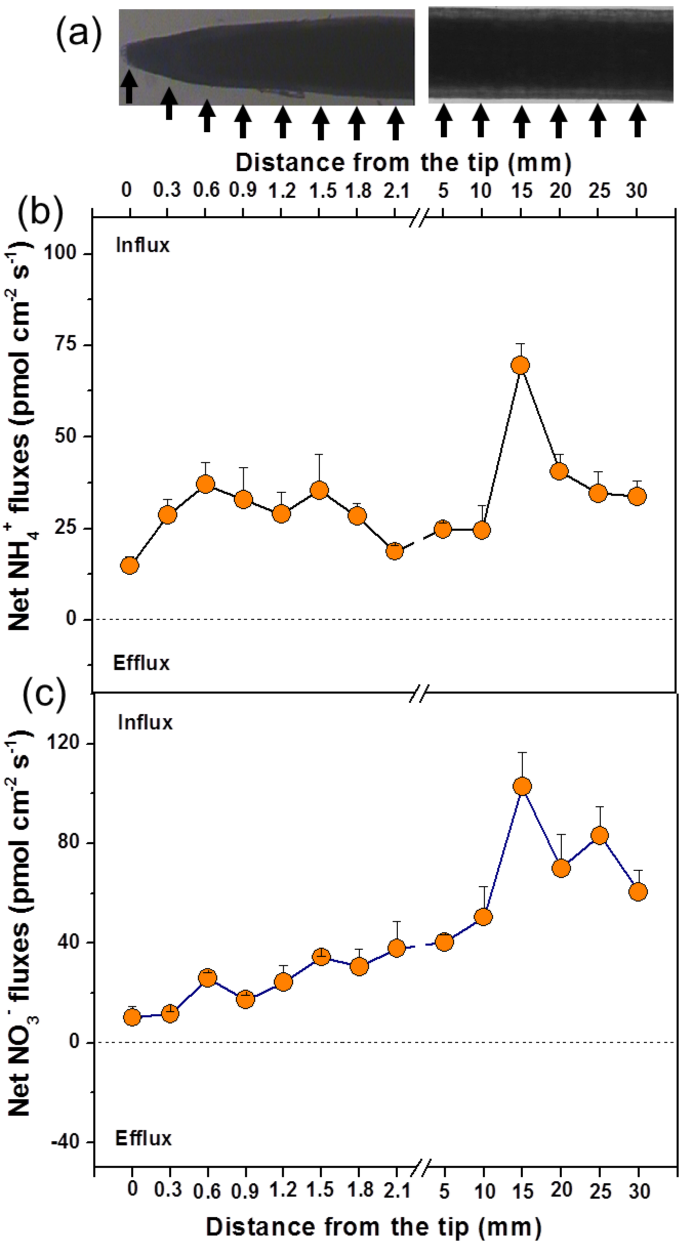

Supplementary Fig. S2. Alignments of representative genes. It is too large to be put here. For detailed information, please see the online version.

Supplementary Fig. S3. NO_2^- contents in roots and leaves of *P. popularis* (Pp) and *P. alba* × *P. glandulosa* (Pg) exposed to 10, 100 or 1000 μM NH_4NO_3. Bars indicate means ± SE (n = 6). Different letters on the bars indicate significant difference. *P*-values of the ANOVAs of species, N treatment (N) and their interaction are indicated. *: *P*<0.05; **: *P*<0.01; ***: *P*<0.001; ****: *P*<0.0001; ns: not significant.

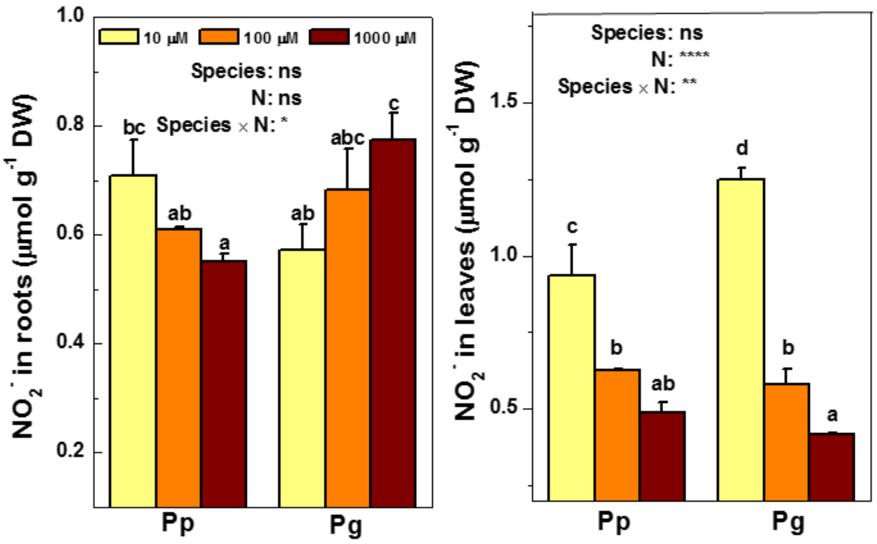

Supplementary Fig. S4. Activities of nitrite reductase (NiR, mmol NO_2^- h^{-1} mg^{-1} protein), glutamate synthase (GOGAT, nkat g^{-1} protein) and glutamate dehydrogenase (GDH, nkat g^{-1} protein) in roots and leaves of *P. popularis* (Pp) and *P. alba* × *P. glandulosa* (Pg) exposed to 10, 100 or 1000 µM NH_4NO_3. Bars indicate means ± SE (n = 6). Different letters on the bars indicate significant difference. *P*-values of the ANOVAs of species, N treatment (N) and their interaction are indicated. *: P<0.05; **: P<0.01; ***: P<0.001; ****: P<0.0001; ns: not significant.

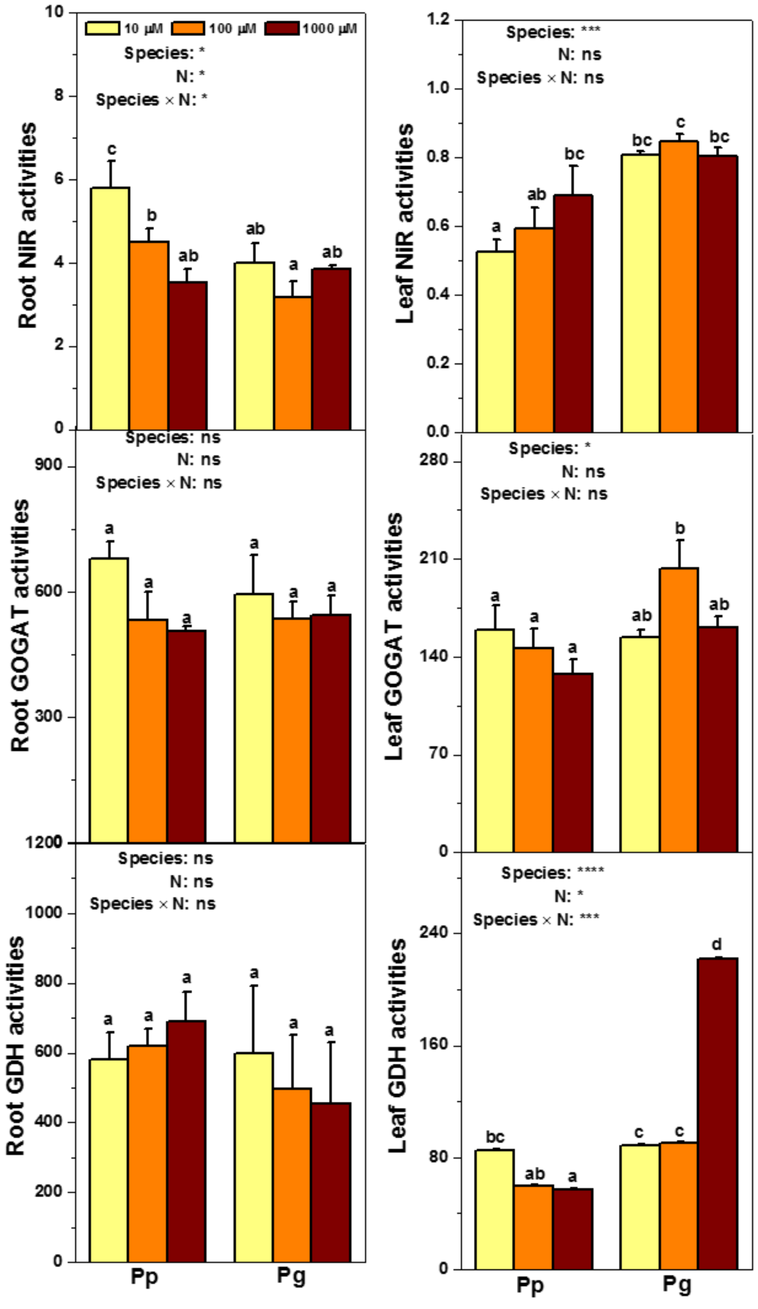

Supplementary Fig. S5. Soluble sugars, C concentrations, $\delta^{13}C$ in roots and leaves of *P. popularis* (Pp) and *P. alba* × *P. glandulosa* (Pg) exposed to 10 (yellow), 100 (orange) or 1000 (wine) μM NH_4NO_3. Bars indicate means ± SE (n = 6). Different letters on the bars indicate significant difference. *P*-values of the ANOVAs of species, N treatment (N) and their interaction are indicated. *: *P*<0.05; **: *P*<0.01; ***: *P*<0.001; ****: *P*<0.0001; ns: not significant.

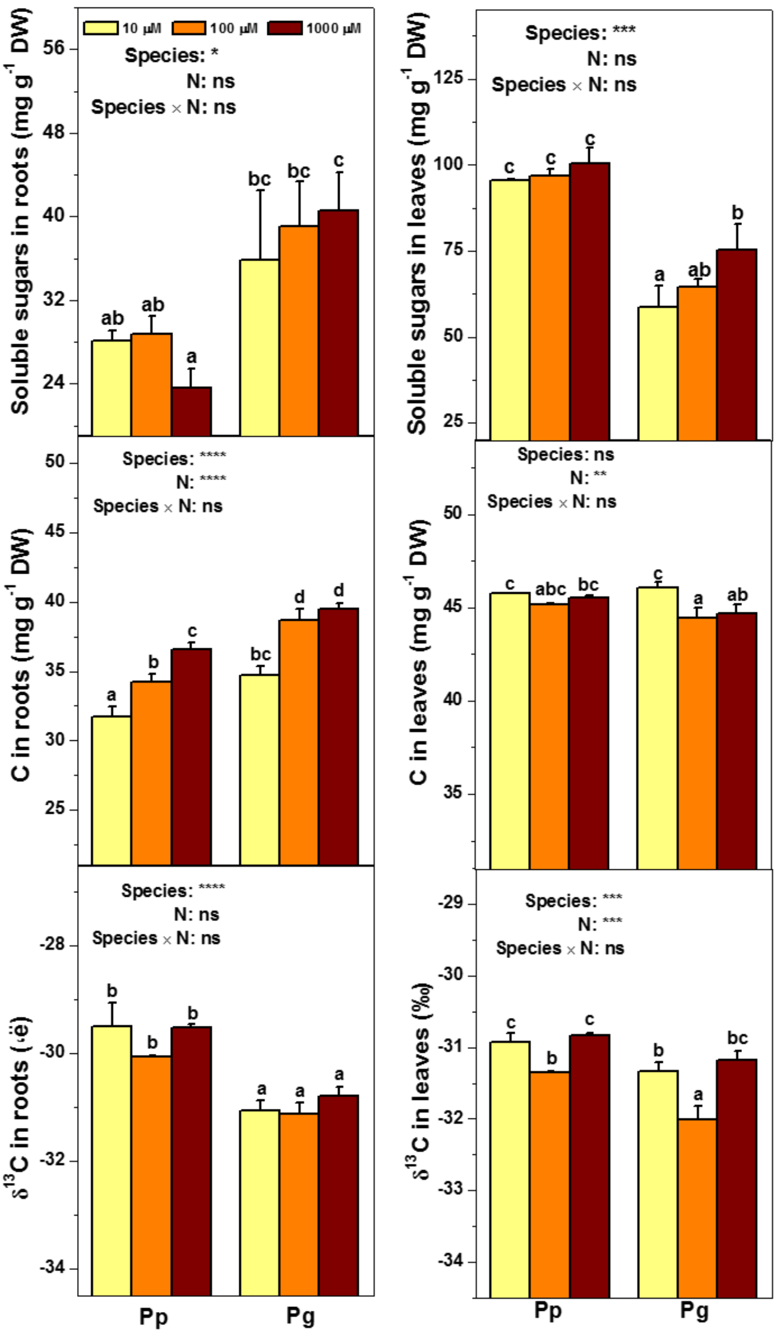

Supplementary Fig. S6. Soluble protein and soluble phenolics in roots and leaves of *P. popularis* (Pp) and *P. alba* × *P. glandulosa* (Pg) exposed to 10, 100 or 1000 µM NH_4NO_3. Bars indicate means ± SE (n = 6). Different letters on the bars indicate significant difference. *P*-values of the ANOVAs of species, N treatment (N) and their interaction are indicated. *: *P*<0.05; **: *P*<0.01; ***: *P*<0.001; ****: *P*<0.0001; ns: not significant.

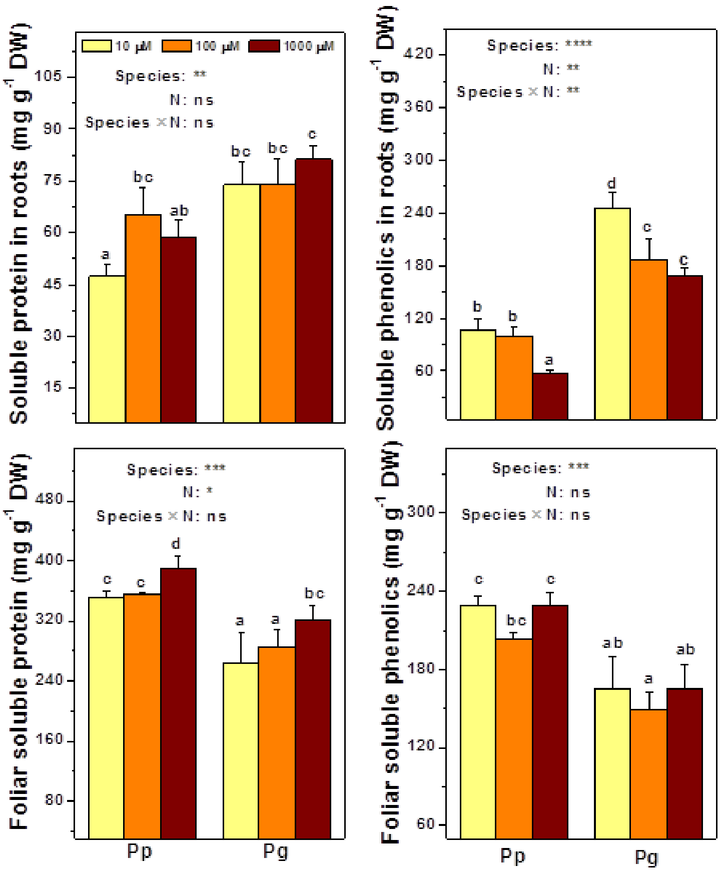

Supplementary Fig. S7. Correlations of related parameters. It is too large to be here. For detailed information, please see the online version.
